# Volatile Organic Compounds in Exhaled Breath as Fingerprints of Lung Cancer, Asthma and COPD

**DOI:** 10.3390/jcm10010032

**Published:** 2020-12-24

**Authors:** Ileana Andreea Ratiu, Tomasz Ligor, Victor Bocos-Bintintan, Chris A Mayhew, Bogusław Buszewski

**Affiliations:** 1 Interdisciplinary Centre of Modern Technologies, Nicolaus Copernicus University, 87-100 Toruń, Poland; andreea_ratiu84@yahoo.com (I.A.R.); tomasz.ligor@umk.pl (T.L.); 2 Department of Environmental Chemistry and Bioanalytics, Faculty of Chemistry, Nicolaus Copernicus University, 87-100 Toruń, Poland; 3 “Raluca Ripan” Institute for Research in Chemistry, Babes-Bolyai University, RO-400294 Cluj-Napoca, Romania; 4 Faculty of Environmental Science and Engineering, Babes-Bolyai University, RO-400294 Cluj-Napoca, Romania; victorbocos@yahoo.com; 5 Institute for Breath Research, Leopold-Franzens-Universität Innsbruck, Innrain 66, A-2020 Innsbruck, Austria; christopher.mayhew@uibk.ac.at; 6 Tiroler Krebsforschungsinstitut (TKFI), Innrain 66, A-2020 Innsbruck, Austria

**Keywords:** analytical platforms, markers of respiratory diseases, lung cancer, chronic obstructive pulmonary disease, asthma

## Abstract

Lung cancer, chronic obstructive pulmonary disease (COPD) and asthma are inflammatory diseases that have risen worldwide, posing a major public health issue, encompassing not only physical and psychological morbidity and mortality, but also incurring significant societal costs. The leading cause of death worldwide by cancer is that of the lung, which, in large part, is a result of the disease often not being detected until a late stage. Although COPD and asthma are conditions with considerably lower mortality, they are extremely distressful to people and involve high healthcare overheads. Moreover, for these diseases, diagnostic methods are not only costly but are also invasive, thereby adding to people’s stress. It has been appreciated for many decades that the analysis of trace volatile organic compounds (VOCs) in exhaled breath could potentially provide cheaper, rapid, and non-invasive screening procedures to diagnose and monitor the above diseases of the lung. However, after decades of research associated with breath biomarker discovery, no breath VOC tests are clinically available. Reasons for this include the little consensus as to which breath volatiles (or pattern of volatiles) can be used to discriminate people with lung diseases, and our limited understanding of the biological origin of the identified VOCs. Lung disease diagnosis using breath VOCs is challenging. Nevertheless, the numerous studies of breath volatiles and lung disease provide guidance as to what volatiles need further investigation for use in differential diagnosis, highlight the urgent need for non-invasive clinical breath tests, illustrate the way forward for future studies, and provide significant guidance to achieve the goal of developing non-invasive diagnostic tests for lung disease. This review provides an overview of these issues from evaluating key studies that have been undertaken in the years 2010–2019, in order to present objective and comprehensive updated information that presents the progress that has been made in this field. The potential of this approach is highlighted, while strengths, weaknesses, opportunities, and threats are discussed. This review will be of interest to chemists, biologists, medical doctors and researchers involved in the development of analytical instruments for breath diagnosis.

## 1. Introduction

Respiratory diseases—including lung cancer, chronic obstructive pulmonary disease (COPD) and asthma—are increasing worldwide. The World Health Organization (WHO) reported that the above-mentioned diseases are associated most often with smoking of tobacco products, a habit that ultimately kills at least 8 million people per year. Moreover, Paul Garwood, Communications Officer of WHO, reported more than 40% of all tobacco-related deaths are due to lung diseases, including cancer, COPD and tuberculosis [1]. While lung cancer is one of the leading causes of death worldwide [2], COPD and asthma are predominant lung diseases that are extremely stressful, limit quality of life, and represent a significant societal costs [3].

Respiratory diseases, especially lung cancer, are often diagnosed only in their late stages, because either of lack of specific symptoms or because they can be confused with transient virus-induced diseases, which delays the chance of applying a timely and effective treatment. The diagnosis procedures for all three diseases are either bronchoscopy, broncho-alveolar lavage or biopsy; all are very invasive, costly and time consuming [4]. Consequently, a non-invasive, fast, inexpensive and reliable screening procedure, realized by means of robust and user-friendly analytical platforms that can replace the classical methodologies for diagnosis of lung cancer, asthma and COPD, is highly required.

Exhaled breath can be the perfect matrix to be investigated. A breath sample is directly connected with the affected organ (the lungs) and may therefore perfectly reveal the emitted endogenous volatiles resulting from oxidative stress. Moreover, owing to its non-invasiveness, patients willingly accept breath sampling.

Other biological matrices, including saliva, breast milk, sweat, epithelial tissue, urine or feces, have been investigated for their use in diagnosing various diseases, for assessing chemical exposure, or for determining drug consumption [5,6,7,8,9,10,11,12].

The investigation of exhaled breath has been studied more intensively compared with other biological samples [13,14,15,16,17,18,19,20,21,22], and for which a number reviews are available in the literature [4,23,24,25,26,27,28,29,30]. However, none of these reviews covers recent studies of VOCs and lung disease undertaken in the last decade.

The focus of this paper is to provide such a review, presented in a systematic way so that the current knowledge on VOC breath biomarkers of lung cancer, asthma and COPD and details on their potential for use as diagnostic tools for lung diseases are provided in one useful source for guiding future studies.

This review will cover studies of lung diseases that have used various analytical platforms for breath analyses [31], including proton-transfer reaction mass spectrometry (PTR-MS) [32,33,34], secondary electrospray ionization—mass spectrometry (SESI-MS) [20], ion mobility spectrometry (IMS) [18,35,36,37], various sensors and E-noses [15,38,39,40,41,42,43,44] and gas chromatography–mass spectrometry (GC–MS) [43,44,45,46,47,48].

GC-MS is regarded as the most selective detection method used in a variety of other areas [7,31,49,50,51,52,53], but requires sample pre-concentration and cannot be used in real-time. Neither PTR-MS, SESI-MS, nor GC-MS can be used in clinical environments at the point-of-care, but all are extremely useful for discovery investigations. For provision of clinical information so that quick and informed medical decisions can be made, IMS type systems and e-noses have significant advantages in terms of their simplicity and low costs.

## 2. Study Design

### 2.1. Articles Selection

A single reviewer (IAR) undertook an extensive literature search covering the years 2010–2019 (literature search was completed on 12 February 2020), using the keywords “VOCs asthma”, “VOCs COPD” and “VOCs lung cancer”, with the following databases being used: Springer, Web of Science, Science Direct and Wiley.

By considering only articles written in English and omitting reviews and book chapters, a total of 2268 papers were identified. Subsequently, by checking the reference list of these selected articles, additional studies were identified and included.

**Figure 1:**
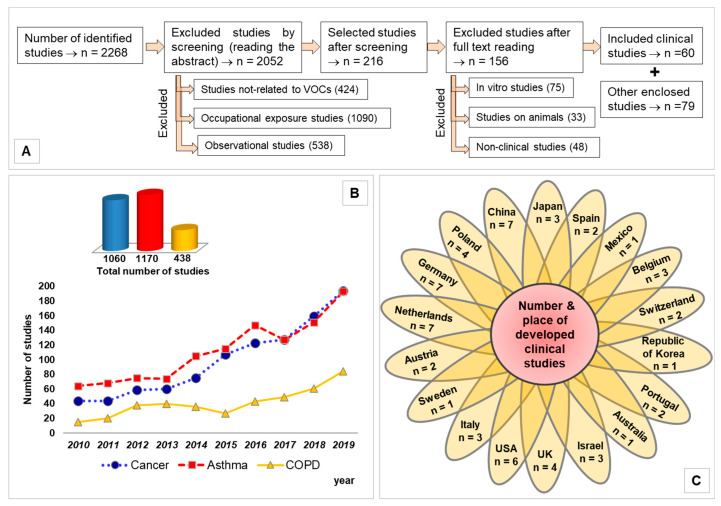
(**A**) Flow diagram presenting the method used for selecting the articles; (**B**) trend line highlighting the number of clinical studies as a function of year; (**C**) flower chart illustrating number of studies by origin of country.

Figure 1A schematically shows the method used for the article selection. Figure 1B illustrates the number of articles found for each category of disease as a function of year, 2010–2019. This shows that the numbers of asthma and lung cancer studies are comparable. However, using a well-defined selection criteria (see next section), the number of articles that are reviewed in this paper for lung cancer is considerably higher than those for asthma. Figure 1C presents the number of studies by country.

### 2.2. Criteria for Selection of Articles

To make this review manageable, articles were excluded using the following criteria:

- No investigation of the VOCs profile, but non-volatile markers;
- Targeted diseases caused by exposure to harmful VOCs;
- VOCs related to the effects of therapy;
- Sampling and/or analyses methods only;
- Sensitivity, specificity, or accuracy of existing methods, with no focus on clinical studies;
- Sensor development used for validation standards of previously reported markers of certain diseases;
- Risk assessment and occupational exposure studies;
- Nanomaterials with application in clinical diagnosis;
- Smoking and/or exposure to tobacco products;
- Predictive models constructed using VOCs targets collected from the literature;
- Non-clinical, in vitro and animals’ studies.

These exclusion criteria dramatically reduced the number of clinical studies to sixty.

### 2.3. Data Structuring

For the sixty clinical studies selected, the following information was extracted: study design, investigated diseases, sampling methods, patient and control characteristics, analytical platform, statistical approach, measured outcomes, identification of VOCs and their quantification (where applicable) and diagnosis performance, e.g., expressed as sensitivity, specificity, accuracy, area under the curve, etc. Owing to the multitude and heterogeneity of the information, the following three tables have been constructed for convenience:

- Table 1 presents details on the type of sample that is collected, participants’ number, and place (hospital, country) where the samples were collected;
- Table 2 summarizes the analytical platforms used, key outputs, statistical approach and diagnosis accuracy;
- Table 3 reports the VOCs that have been identified to be associated with the three respiratory diseases.

## 3. Statistics of Included Studies

A total of 139 studies were included in the present review. From the total of 60 cross-sectional clinical studies selected [13,14,15,16,17,18,19,20,21,22,32,33,34,35,36,37,38,39,40,41,42,43,44,45,46,47,48,54,55,56,57,58,59,60,61,62,63,64,65,66,67,68,69,70,71,72,73,74,75,76,77,78,79,80,81,82,83,84,85,86], 33 are related to lung cancer [13,15,16,21,22,33,34,36,41,44,48,56,58,59,60,62,64,66,67,68,69,70,71,72,73,74,75,77,78,80,82,84,86], 14 are associated with COPD [14,18,19,20,32,35,37,39,54,57,63,79,81,85], and 10 present details on asthma [17,42,43,45,46,47,55,61,65,76]. Two other studies presented information on how to discriminate between patients with COPD or asthma [38,40]. One other study reported details on discriminating lung cancer from COPD [83]. A total of 7072 participants formed two main groups: one group is for patients diagnosed with one of the three diseases investigated (3478) and healthy controls (3132), totaling 6610 subjects. The difference is made by a study that investigates 462 participants without mentioning the number for each category [86]. One study [86] presents the reanalysis of data previously reported [87]. Within these groups, 1601 participants were involved in COPD studies (846 patients and 755 controls); 845 volunteers were involved in asthma studies (614 patients and 231 controls), and the largest number of participants at 4626 were associated with lung cancer studies (2053 patients, 2111 controls and 462 unknown).

Smokers have volatiles in their breath that result in confounding biomarkers and, hence, these must be taken into account. For lung cancer patients, 601 participants reported to be active smokers, 602 were former smokers and 328 never smoked. For COPD, 257 people were active smokers, 361 former smokers and 62 never smoked. For asthma, 5 were declared to be active smokers, 52 were former smokers and 38 never smoked. Concerning the smoking status of the controls, a total number of 847 were active smokers, 395 were former smokers and 936 never smoked. The differences between the total number of patients and smokers is because the smoking status was not revealed in all of the papers, but also because a number of the studies (especially those related to asthma) involved children. From the total number of participants, 421 were children with 229 children having asthma and 192 children acting as the controls. Of the selected clinical studies, thirty-two of them reported that they used mixed expired breath [13,17,19,21,33,34,36,38,41,42,43,45,46,54,59,60,61,62,64,66,67,68,69,71,72,73,75,76,78,80,83,85] (consisting normally in a mixture of gaseous breath, that also includes the volatile components) collected by simple expiration in bags, tubes with absorbent materials or directly into the used instrumentation (as in the case of E-noses, for example). A total of twenty-two of them reported the use of alveolar breath [14,15,16,18,22,32,35,37,44,48,56,57,58,65,70,74,77,79,81,82,84,86] (collected at the appropriate time by monitoring CO_2_ levels as a function of time). Three studies collected exhaled breath condensate [20,39,40] (all for COPD investigations). One study collected both mixed and alveolar breath [55] and two studies examined mixed breath plus sputum [47,63]. The clinical studies included in this review were undertaken in 18 different countries. The information summarized above is presented in more detail in Table 1.

## 4. Analytical Platforms Used for Investigating Breath Volatiles Associated with Asthma, COPD, and Lung Cancer

Several analytical spectrometric techniques can be used for analyzing volatiles contained in exhaled breath samples. When choosing an analytical method, many aspects need to be considered, including the advantages and disadvantages of a particular analytical technique, and whether offline or on-line sampling is needed. Below, we describe the key analytical instruments that have been used to investigate breath volatiles and lung diseases.

### 4.1. GC-MS Instrumentation

For offline measurements, GC-MS is the most powerful tool, with a high sensitivity (sometimes lower than ppb range) and, more importantly, a high potential for both identification and quantification of unknown components from complex biological matrixes [4,8,9,10,88,89]. Moreover, by using different columns and detectors a great versatility in targeted analyses can be achieved [90,91].

Owing to its size and length of analysis (tens of minutes to hours) GC-MS cannot be used at clinical points of care, even if, at the research level, GC-MS remains the gold standard for VOC analysis in many fields [92,93,94,95]. GC-MS analysis requires the samples to be collected, either in special bags or onto absorbent materials, and then transported to the laboratories, resulting in samples being stored for days and even weeks before analysis. Of the 60 clinical studies being reviewed in this paper, 29 used various types of GC-MS systems. Two groups used two-dimensional GC, explicitly GC×GC-FID [69] and TD-GC×GC-ToF-MS [13] for lung cancer investigations. Caldeira and co-authors [46] used TD-GC×GC-ToF-MS to investigate exhaled breath metabolomes of patients with allergenic asthma.

### 4.2. PTR-MS and SESI-MS Instrumentation

PTR-MS and SESI-MS can be, and have been, used offline to analyse breath samples, but they come into their own for online analysis. However, the advantages of real-time analysis, which allows rapid changes in volatile concentrations to be detected, comes at the expense of identifying the volatiles with a high level of confidence [96,97,98,99]. Nevertheless, the near patient analyses mean that samples do not need to be transported and hence storage is not necessary. Consequently, deterioration of the breath samples and storage errors are avoided. As for GC-MS, PTR-MS, and SESI-MS require skilled operators. Generally, the cost of a PTR-MS, and particularly PTR-ToF-MS, being between EUR 200,000 and 500,000 are far more than the cost of GC-MS instruments (EUR 60,000–150,000) and, hence, there are fewer PTR-MS studies compared to GC-MS. Although the cost of a SESI-MS is lower than that of a GC-MS, it has only been rarely used. PTR-MS was used in two studies of lung cancer [33,34], and one study for discriminating COPD and emphysema [32]. SESI-MS was involved in a single study for COPD diagnosis [20]. Another soft chemical ionization mass spectrometric technique that could be used in real-time for discovery programmes is the Selected Ion Flow Tube Mass Spectrometry but, to our knowledge, no study of breath volatiles and lung disease involving this instrument has been reported. No study is presented for SIFT-MS.

### 4.3. IMS Based Instrumentation

Another category of analytical instrumentation suitable for VOCs analysis in real or near to real time is ion mobility spectrometry (IMS), both as a standalone tool and coupled with GC columns that provide a pre-separation. The costs of instrumentation are considerably lower than the previous mentioned techniques based on mass spectrometry (ranging from between EUR 7,000 and 30,000 for standard IMS, while GC-IMS can range between EUR 50,000 and 60,000). That no vacuum system is required dramatically reduces the size and power requirements. Together with its ease of use and robustness, IMS, and particularly GC-IMS, is extremely suitable for use in clinical environments at the point of care [100,101,102,103,104]. The most common types are the classical IMS, a-IMS (aspiration IMS), FAIMS (Field Asymmetric wave IMS) and DMS (differential mobility spectrometry). For improved analytical dimensionality, GC-IMS and MCC-IMS (multi-capillary column IMS) are also used [91]. Amongst the clinical studies that we review, MCC-IMS has been used to investigate patients with COPD [18,35] and lung cancer [36,74]. One other study used a double approach by comparing GC-IMS and GC-APCI-MS (atmospheric pressure chemical ionization MS) for investigating breaths samples from patients with COPD [37].

### 4.4. Sensors and Electronic Noses

Analytical instrumentation related to online measurements also comprises simple sensors and electronic noses (e-noses). They are usually cheap, easy to operate and have the capacity of real-time monitoring based on pattern recognition algorithms. Moreover, they are often equipped with software that compares VOCs-emitted profiles of ill patients with those of healthy individuals [15,64,105]. Their main drawback is their lack of selectivity, VOCs are not identified, reproducibility may be affected by interferences, thereby diminishing the reliability, and robustness.

E-noses were successfully applied in discriminating exhaled air of patients with asthma from healthy controls; a commercial system model Cyranose 320, consisting of an array of 32 organic polymer sensors, has been used [106]. The same nanosensor array (Cyranose 320) has been utilized for discriminating patients with lung cancer and COPD, when it has been shown that an electronic nose is able to distinguish the VOCs pattern in exhaled breath of lung cancer patients from healthy controls; the authors pointed out in a realistic manner that, although the electronic nose may become a very convenient tool for a physician, this instrument may qualify as either a screening tool or a pre-diagnostic tool by selecting patients for further diagnostic and testing procedures [107]. Analysis of exhaled VOCs in order to discriminate COPD phenotypes, using a Bionote electronic nose (comprising of a seven quartz microbalance (QMB) sensor array, with the sensors being covered with anthocyanins that are used as chemical sensitive materials), has been described in several original research papers [108,109].

Application of e-noses and other types of sensors to breath analysis has been addressed by a review focusing to methodological issues related to applying e-noses to breath analysis. Although they possess strong capabilities in rapidly discriminating samples of exhaled breath (the so-called “breathprint”), the e-nose is not currently ready for point-of-care use [110].

Another valuable review summarizes the role electronic noses play in distinguishing different endotypes by using VOCs in exhaled breath; breath sampling and metabolism of VOC biomarkers are also summarized [111].

Of the 60 clinical studies included in this review, nine studies used sensors or e-noses [15,38,39,40,42,64,66,77,83], while five studies used both sensors or e-noses and an additional GC-MS (or a related) technique as a confirmation method [21,55,58,78,85]. For example, Cyranose 320 (Smiths Detection, Pasadena, CA, USA) e-noses were used to discriminate between asthma and COPD [38,40]; another type of e-nose, Aeonose (The eNose Company, Zutphen, The Netherlands) was utilized to differentiate between children with asthma and cystic fibrosis [42]. The Cyranose 320 system is a portable device that incorporates 32 chemical sensors that provide a different response to various VOC mixtures; these chemiresistor sensors are made from carbon black nanocomposites that have the ability to change their resistance as a response to VOC exposure [39]. Aeonose is an easy-to-use hand-held e-nose, weighing just 650 grams, equipped with three metal-oxide sensors, which behave as semiconductors at higher temperatures [42].

In terms of other sensors, colorimetric sensor array [64], metal oxide gas sensors [15] and nanosensors based on organically functionalized gold nanoparticles [58] have been used to investigate their potential for use in cancer diagnosis.

### 4.5. Fourier-Transform Ion Cyclotron Resonance Mass Spectrometry

Fourier-transform ion cyclotron resonance mass spectrometry (FT-ICR-MS) is an analytical technique that can be used for targeted detection and quantification of VOCs. Using *“a hybrid linear ion trap Fourier transform (FT) ion cyclotron resonance (ICR) mass spectrometer (MS) equipped with a TriVersaNanoMate ion source with an electrospray chip (nozzle inner diameter 5.5 mm)”* researchers claim to have identified specific carbonyl cancer markers (mainly 2-butanone, 3-hydroxy-2-butanone, 2-hydroxyacetaldehyde and 4-hydroxyhexenal) that can differentiate benign pulmonary disease from early-stage lung cancer [67,68,71,73].

### 4.6. Trained Dogs

It is worth mentioning that trained dogs have been used to “sniff” for diseases, with claims of good performances being apparently comparable, if not better, to various analytical devices. In two studies included in the present review, trained dogs were used [44,83], while a new article related to two-step investigation of lung cancer detection, where the abilities of sniffer dogs were proved in maintaining their discriminative capacity under long-term, and in different types of environments, appeared after the articles’ collection period closed [112].

### 4.7. Features and Performance of Analytical Platforms

**Figure 2:**
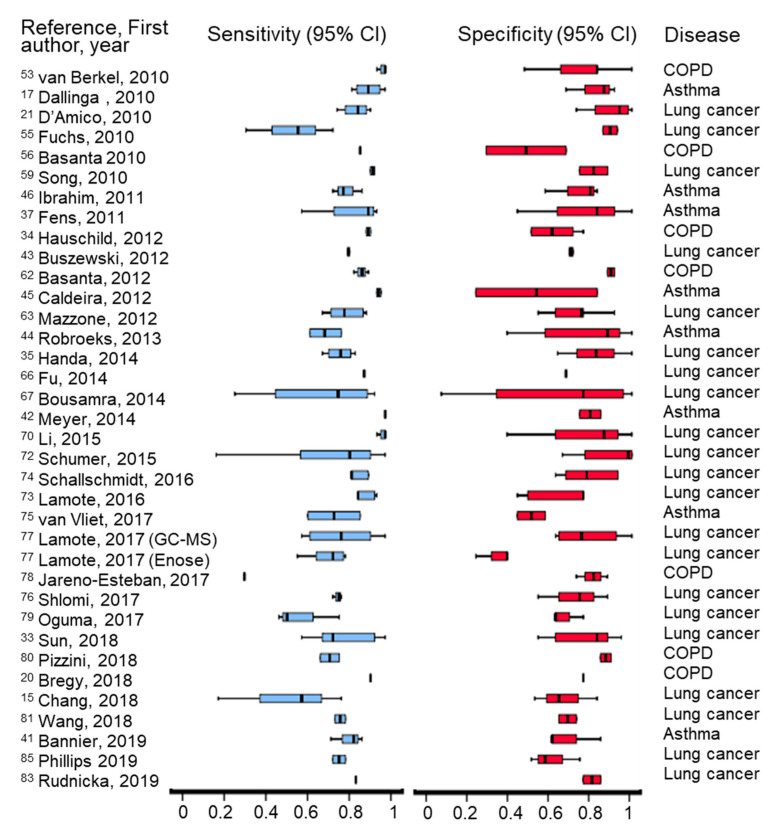
Graphical representation of recorded sensitivity and specificity obtained in the clinical studies included in the review, by using different analytical techniques.

All the clinical studies reviewed in this paper describe various methods of optimization at different levels (sampling, analysis, data processing and interpretation, etc.) in order to enhance diagnostic capabilities. A summary of sensitivity and specificity obtained by different studies is presented in Figure 2. Multiple statistical approaches have been used to classify the detected VOCs using different models. Details about each clinical study, including analytical platforms, statistical approaches, and outcomes are presented in Table 2.

## 5. Diagnosis of Investigated Respiratory Diseases

### 5.1. Asthma

Asthma is a chronic inflammatory condition, which produces reversible airways obstruction, often beginning in childhood, and characterized by triggering bronchospasms. The common symptoms include short episodes of chest tightness, wheezing, coughing, and a shortness of breath, with these symptoms being in some people more pronounced during the night or following strenuous physical exercises [4,17,113]. Although appearing from partially unknown causes, it is considered that asthma is often caused by environmental pollution, irritant agents, allergens (pollen, dust, fur etc.) or drugs (aspirin and beta blockers) [114]. Both asthma and COPD diagnosis is based on symptoms, long term response therapy lung capacity tests, and spirometry tests, which includes: (1) FVC (forced vital capacity): largest volume of air that can be forcefully exhaled and (2) FEV (forced expiratory volume): how much air can be exhaled in one second) [115]. Gastroesophageal refluxes, eosinophilia, neutrophilia, allergic rhinitis, obstructive sleep apnea and atopy are conditions frequently occurring in people with asthma [27,47]. The atopy (the triad of asthma, allergic rhinitis and eczema together) is the predisposition towards developing hypersensitivity reactions and triggering exacerbations. An exacerbation is an asthma attack crisis that may also appear in non-atopic asthmatics. Asthma cannot be cured; the prevention includes avoiding the allergenic and irritants agents and the use of inhaled corticosteroids. In 2015, 358 million people were globally registered as diagnosed with asthma, with 397,100 deaths attributed to the disease [2,3].

#### Diagnosis of Asthma Based on Specific VOCs

The main disadvantage of traditional tests used for diagnosing asthma resides in the fact that they are time consuming and some of them are invasive. Both invasive and non-invasive (spirometry and fractional exhaled nitric oxide) diagnostic techniques are used. However, non-invasive diagnosis based on exhaled VOCs is promising, and hence has recently been gaining increasing attention. In eight studies, asthma diagnosis was tested using GC-MS analysis. For example, Dallinga et al. [17] analyzed the breath samples of 63 asthmatic children and compared them to breath samples from 57 healthy controls (5 to 16 years old). Only eight VOCs were found to be needed to discriminate diseased from healthy children (with a claim of 92% correct classification, a sensitivity of 89% and a specificity of 95%) [17]. A set of eight compounds was used in another study to discriminate between healthy and asthmatic children; however just one of them, 2-octenal, was proposed as a certain marker of asthma, because the authors concluded that the others may have other possible origins [65].

The ability to diagnose allergenic asthma—sometimes combined with allergic rhinitis in children—was tested using HS-SPME/GC–qMS and a comprehensive two-dimensional GC×GC–ToF-MS [46,61]. Almost similar statistical tools were involved for data processing, and the two-dimensional GC×GC–ToF-MS proved its superiority in comparison to GC-MS. In the first study by Caldeira et al., [61] a set of 28 VOCs was used to discriminate between asthmatic and control group, with a classification rate of 88% [61]. In their second study [46], a pattern of just six chemicals, namely nonane, 2,2,4,6,6-pentamethylheptane, decane, 3,6-dimethyldecane, dodecane, and tetradecane, were used, with a classification rate of 98% being achieved, with 96% sensitivity (meaning that only ∼4% allergic asthma children were misclassified as controls) and 95% specificity (meaning only ∼5% were classified as false positives). All six chemicals were, therefore, proposed as biomarkers of asthma [46]. Exacerbations in case of atopic asthmatics children were predicted based on emitted VOCs analyzed by GC-MS [45,76]. In the first study, the applied classification model used seven VOCs that provided a correct classification rate of 91% for those patients, who experienced exacerbations (sensitivity of 79% and specificity 100%). Moreover, they demonstrated that the FeNO and lung function were not predictive for exacerbations [45]. The classification model used in the second study was based on seven selected VOCs, three aldehydes, one hydrocarbon, one ketone, one aromatic compound, and one unidentified VOC, which achieved a sensitivity of 88% and a specificity of 75%, with AUC of ROC 90% [76]. Electronic noses were used for discrimination between asthma and COPD, asthma and cystic fibrosis, and for asthma diagnosis [38,40,42].

Aeonose, a patient-friendly and easy to use e-nose device, was utilized to test the discrimination and diagnostic accuracy for children with asthma and cystic fibrosis. The reported mean values for discrimination between asthma and cystic fibrosis were as follow: AUC = 0.90 (95% CI), sensitivity 89%, specificity 77%, while for differentiation between healthy controls and cystic fibrosis the mean scores were slightly lower: AUC = 0.87, sensitivity 85% and specificity 77%. However, the authors reported that diagnostic accuracy in the case of asthma and healthy controls discrimination was lower compared with the first two cases (AUC = 0.79, with a sensitivity of 74% and specificity 91%) [42]. A Cyranose 320 instrument was also used discriminate between asthma and COPD in two studies [38,40]. Consequently, an 88% accuracy for distinguishing between asthma and COPD was obtained in the first study [38]. In the second study, two groups (asthmatics and COPD patients), both with and without gastro-esophageal reflux disease (GORD) were investigated, in an attempt to distinguish patients with GORD from those without. The discrimination between patients with COPD, with and without GORD, achieved an accuracy of 67.6%, while the asthmatic group with GORD was differentiated from asthmatics without GORD with an 85% accuracy.

### 5.2. COPD

COPD can coexist with asthma and can actually occur as a complication of chronic asthma. Generally, after the age of 65, most people with asthma will also develop COPD. In this setting, COPD can be differentiated by increased airway neutrophils, abnormally increased wall thickness, and increased smooth muscle in the bronchi [116]. Although having most of the common symptoms of asthma, unlike asthma, COPD is a progressive disease characterized by sputum production and irreversible airways obstruction, which does not improve much with the use of bronchodilators [116]. The most common cause of COPD is tobacco smoking [115,116]. In 2015 only, COPD globally affected about 174.5 million people and it resulted in 3.2 million deaths [2,3].

#### Diagnosis of COPD Based on Specific VOCs

The COPD diagnosis is almost similar to that of asthma, while a VOC analysis is also possible. COPD was investigated by GC-MS in six studies included in this review [14,19,54,63,79,81]. Phillips and co-authors involved 119 patients with COPD and 63 controls in their study. Machine learning approaches were used and models were automatically generated, which correctly predicted the diagnosis in 64% of controls and 79% of patients, obtaining an AUC of ROC of 0.82 [14]. Better discrimination was obtained by Van Berkel et al., [54] who used six VOCs that correctly classified 92% of the subjects with a sensitivity and specificity of 98 and 88%, respectively. Moreover, 14 out of 15 steroid-naïve patients were also correctly classified [54]. Besides discriminating between COPD group and healthy controls, the identification of various COPD subgroups has also been achieved [63]. Notwithstanding, Pizzini and al. went into more details and succeeded to perform differential diagnosis between patients with COPD and COPD with acute exacerbations—a complication caused by infectious and non-infectious agents [81].

It is widely acknowledged that smoking results in respiratory disease development, including COPD. In support of this, Gaida and co-authors developed a dual center study in order to compare the VOCs emitted by smokers and non-smokers, with the volunteers having or not having COPD [19]. Their results highlighted that active smokers are clearly discriminated from the non-smokers. Furthermore, by characterizing 134 VOCs, they were able to provide evidence for 14 VOCs related to COPD.

Real time SESI-HRMS (Secondary Electrospray Ionization—High-Resolution Mass Spectrometry) was used as a diagnostic tool for COPD. A number of 1441 different VOCs were identified, but only 43 were used to discriminate between groups, obtaining an accuracy of 89%, a sensitivity of 93% and a specificity of 86% [20].

PTR-MS was utilized to explore breath samples of heavy smoker patients with emphysema [32], patients who are at risk to develop COPD, based on the hypothesis that emphysema is defined by airways inflammation that alters the composition of the exhaled air. Even if the authors reported that in COPD/emphysema screening the proposed method did not provide a valuable diagnostic tool, a series of VOC markers associated with this disease are presented [32].

A multi-capillary column (MCC-IMS) was used to diagnose COPD in comparison with COPD plus bronchial carcinoma (BC). The statistical learning methods applied were able to distinguish between the patients groups. Healthy and COPD groups were discriminated with a 94% accuracy, while BC on COPD/no-COPD was classified with a 79% accuracy [35]. Besa et al. also used MCC-IMS to differentiate COPD patients from healthy subjects. A number of 137 spectral peaks proved to be statistically significant between the COPD, healthy smokers and nonsmoker groups, while just six VOCs correctly discriminated the COPD patients from healthy controls with a 70% accuracy [18]. Moreover, 15 peaks discriminated between healthy smokers and healthy nonsmokers [18]. A prototype of a compact, closed gas loop GC-IMS was developed and used in an attempt to find correlations between volatiles from COPD patients and controls [37]. A second approach was made to provide a comparison between the results obtained and those acquired by using a modified mass spectrometer with atmospheric pressure chemical ionization with GC pre-separation (GC-APCI-MS). In the case of GC-IMS, three VOCs highlighted significant differences between the COPD and healthy groups, while in the case of GC-APCI-MS, one distinctive VOC, 2-pentanone, has been identified as a COPD specific marker [37].

Ultrafast gas chromatography equipped with an electronic nose detector (FCG eNose) has been used to discriminate between patients with COPD and healthy controls, using a set of 17 VOCs, which correctly classified the groups with an 82% accuracy, 96% sensitivity and 91% specificity [85]. Hattesohl et al. used a Cyranose 320 eNose instrument to measure VOCs patterns of patients with COPD with and without alpha 1-antitrypsin (AAT) deficiency [39]. These authors proved that an e-nose system can differentiate VOC prints of COPD patients with AAT deficiency by obtaining a cross-validation value of 82% (with a sensitivity of 100% and a specificity of 100%) when exhaled breath condensates of AAT-deficiency and COPD groups were compared. In pure exhaled breath, the cross-validation value was lower, being just 58.3% (with a sensitivity of 1.00 and a specificity of 1.00).

### 5.3. Lung Cancer

Malignant tumors, which are formed due to uncontrolled cell growth tissues localized in the lungs, are defined as lung cancers. The most common symptoms that could predict the onset of lung cancer are coughing, a shortness of breath, a pain into the chest and weight loss. It is considered that about 85% of lung cancers are caused by tobacco smoking, with the remaining maximum 15% of cases resulting from exposure to radiation, radon, asbestos, and various forms of air pollution. Other causes result from passive smoking or genetic factors [117].

#### 5.3.1. Types of Lung Cancer

**Figure 3:**
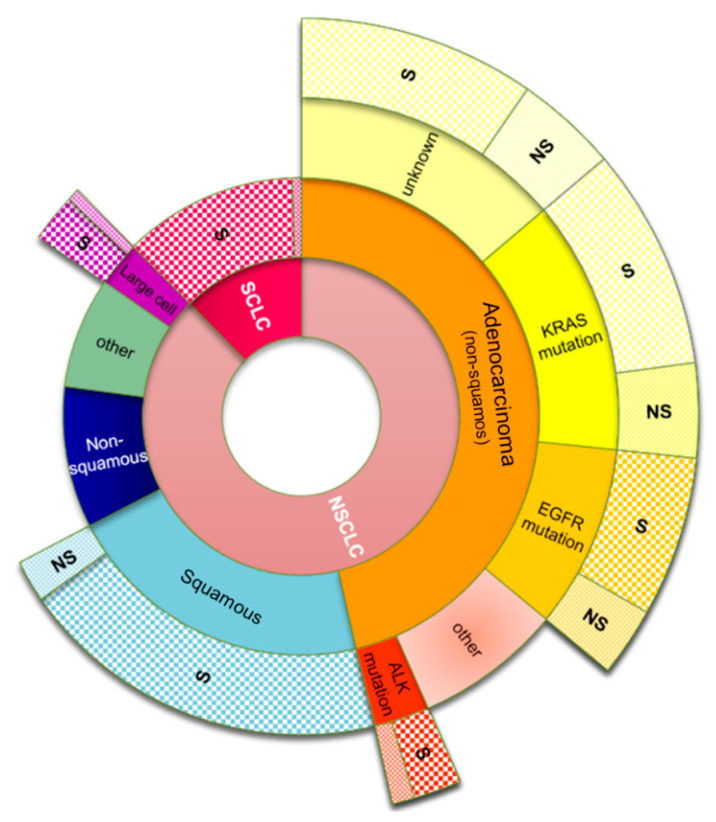
Multistep classification of lung cancer subtypes; common mutations that occur in adenocarcinoma tumors and the occurrence ratio caused by smoking tobacco for each subtype. Abbreviations: SCLS: small-cell lung carcinoma; NSCLC: non-small-cell lung carcinoma; EGFR: epidermal growth factor receptor; KRAS: Kirsten rat sarcoma; ALK: anaplastic lymphoma kinase; S: smokers; NS: non-smokers. Data adapted from Zago et al., Naidoo et al., Kenfield et al. [119,120,121,122].

The primary lung cancers are known as carcinomas (LC) that, according to the histological type, belong to two main categories: small-cell lung carcinoma (SCLC) and non-small-cell lung carcinoma (NSCLC). SCLC consists of dense cells containing neurosecretory granules in the form of blisters full of neuroendocrine hormones; a reason why these kinds of tumors are associated with endocrine or paraneoplastic syndromes. SCLC accounts for about 15% of all lung cancer worldwide [118]. NSCLC accounts for approximately 85% of lung cancers. The most common types of NSCLC are squamous cell carcinoma, non-squamous cell carcinoma (which include adenocarcinoma), large cell carcinoma, and several other types that occur less frequently. The most frequently appearing is adenocarcinoma, located generally peripherally in the lungs [119]; this form of LC accounts for approximately 40% of all lung cancers [120]. Molecular testing allows for possible mutations in the adenocarcinomas to be identified; the most frequently appearing mutations are summarized in Figure 3.

Squamous cell carcinomas tend to be centrally located in the lungs; they are more common in men than in women, and are mostly associated with smoking [122]. Large cell carcinoma is a malignant neoplasm composed of large tumor cells resulting from transformed epithelial cells in the lungs. It can be differentiated from squamous cell carcinomas and adenocarcinomas by light microscopy [123].

#### 5.3.2. Diagnosis of Lung Cancer Based on Specific VOCs

Lung cancer is often diagnosed by chest radiographs or by computed tomography; however, the diagnosis needs to be confirmed by biopsy, which is an invasive, time consuming and expensive diagnosis method with risks. Therefore, many lung cancer studies of breath VOCs have been undertaken in the hope to discover breath biomarkers of the disease and thereby realise a simple non-invasive test. However, despite intense work, to date, no breath test for lung cancer has been forthcoming. A major reason for this is that there has been little consensus between studies, with limited agreement as to which breath volatiles (or pattern of volatiles) can be used to discriminate people with lung cancer from those without. Although many breath volatiles have been proposed to result from lung cancer, not a single study, thus far, has specifically pinpointed the origins of the breath volatiles exclusively to lung cancer nodules and not oxidative stress in any other organ resulting from cancer or any other disease.

In the research related to the diagnosis of lung cancer, GC-MS has been widely used, accounting for more than 50% of studies. In the 15 studies included in this present review, GC-MS was used for analyses [13,16,22,44,48,60,62,69,70,72,75,80,82,84,86], while in another three studies, GC-MS was used as an additional confirmatory method [21,58,78].

Two studies used an ingenious sampling method, SPME-OFD (Solid Phase Micro-Extraction On-Fiber Derivatization), followed by GC-MS analysis, for detection of targeted aldehydes (biomarkers of oxidative stress), which were previously transformed in stable oximes by means of SPME-OFD [56,59]. Exhaled aldehydes C_1_–C_10_ [56] and C_3_–C_9_ [59], respectively, were detected.

Different statistical approaches and machine learning algorithms have been used in order to classify the samples analyzed by GC-MS, coming from patients with lung cancer and from healthy controls [48,64,70,71,74,86]. In an attempt to get closer to a standardization of lung cancer diagnosis, Kischkel et al. applied five different algorithms to process their GC-MS data [48]. Their results concluded that exhaled VOCs are dependent on a multitude of factors, other than the investigated diseases (i.e., patients’ medical history, environmental conditions) [48]. 

GC-MS profiles of potential markers of lung cancer were investigated in four different studies by a Polish group [22,44,62,84]. They carried out qualitative and quantitative measurements by sampling human breath using solid phase SPME and gas chromatography—time-of-flight mass spectrometry (GC–TOF/MS), obtaining possible biomarkers (19 to 32 VOCs) at the level of parts per billion, when more subtypes of lung cancer were investigated (SCLC, NSCLC, adenocarcinoma, planoepitheliale, squamous cell carcinoma). Sons et al. [60] used GC-MS to investigate two types of lung cancer: adenocarcinoma and squamous cell carcinoma, covering all four stages of the disease, and proposed just two key volatile biomarkers that were found at significantly higher concentrations in the breath of the lung cancer patients compared to the controls: 1-butanol and 3-hydroxy-2-butanone (acetoin). For 1-butanol, the obtained AUC was 0.940, with a sensitivity and specificity of 0.953 and 0.854, respectively, while for acetoin, the AUC was 0.964, whereas the sensitivity was 0.930 and specificity 0.927. Moreover, other important conclusions were revealed: higher concentrations of both targets were found in adenocarcinoma than in squamous cell carcinoma, and the concentrations of the VOCs could not be correlated with the stage of disease [60]. Adenocarcinoma and squamous cell carcinoma subtypes were discriminated in a PTR-MS study. The authors claim that breath volatiles from adenocarcinoma and squamous cell carcinoma patients can help in identification of cancer subtypes [34].

Three types of lung cancer (adenocarcinoma, squamous cell carcinoma, and small cell carcinoma) that were histologically proven were analyzed using MCC-IMS, with the obtained VOC profiles were compared with a healthy control group. In addition, adenocarcinoma samples, with and without epidermal growth factor receptor (EGFR) mutation, were also compared. The decision tree algorithm used was able to discriminate the groups of patients based on the 115 detected VOCs. Moreover, n-dodecane was found to be significantly higher in 14 patients with EGFR mutation than in those negative for EGFR (*p* = 0.01). The applied decision tree algorithm differentiated therefore the positive EGFR samples from those negative with a sensitivity of 85.7% and a specificity of 78.6% [36]. Almost similar results were obtained by Shlomi and co-authors, who discriminated patients with EGFR mutation from other groups investigated with 83% accuracy, while the sensitivity and specificity were 79% and 85%, respectively. For samples analyses, a highly sensitive nanoarray of sensors, containing 40 cross-reactive chemically diverse chemiresistors, was used [77].

The interference of benign pulmonary diseases (BPD) in the selection of VOCs markers for lung cancer has been reported [82]. SPME and TD (thermal desorption) were used together with GC-MS to classify four groups of samples: from patients with lung cancer, from patients with BPD, the group with lung diseases (including lung cancer and BPD) and the group of healthy controls. The main scope was to check if the benign lung tumors led to the generation of VOCs that interfere with those considered to be associated with lung cancer. The authors concluded that the discrimination between the lung cancer group and the control group, and between the BPD group and the control group, is possible with an accuracy of 70–80%. However, no VOCs could discriminate between the lung cancer group and BPD group [82]. A kindred study was developed by Zou et al. [70], where the breath samples coming from 171 volunteers divided into three groups (with lung cancer, with BPD and controls) were analyzed by GC-MS. They suggested that five detected volatiles are associated with lung cancer. They reported that they succeeded in discriminating the three preselected groups, avoiding the interference between lung cancer and pulmonary non-malignant diseases [70]. However, only an AUC higher than 0.80 can state a good predictability of diagnosis [10]; consequently, the authors obtained good diagnosis accuracy just in case of one volatile (AUC = 0.84), but satisfactory to low in case of the other four VOCs reported (AUC = 0.67 to 0.78). Moreover, by applying PCA, a partial discrimination of lung cancer group from control and BPD group was obtained [70]. Good discrimination of lung cancer from benign nodules (with an 87% accuracy) was obtained in another study, by using an electronic nose system consisting of highly sensitive nanoarray sensors [77]. In addition, discrimination feasibility between BPD and lung cancer was proved in other four studies [67,68,71,73]. The authors succeeded in proving a good diagnosis prediction for lung cancer, avoiding the BPD interferences, when FT-ICR-MS was used for analyzing breath samples.

Feinberg et al. used PTR-MS to study volatile fingerprints in the exhaled breath of patients with lung cancer before and after an oral glucose tolerance test, to investigate whether tumor cells hyper-glycolysis can affect the volatile signatures [33]. The authors concluded that oral glucose tolerance test has a minimal effect on the VOC profile of patients group, while the profiles coming from the control group were significantly changed after the induced hyper-glycolysis. It was proposed that this is due to the ceiling effect present in cancerous patients [33].

Malignant Pleural Mesothelioma (MPM, which is predominantly caused by asbestos exposure) was investigated using MCC-IMS. Discrimination of MPM patients from control groups was achieved with an overall accuracy of 76%, a ROC-curve of 0.81, an 87% sensitivity and a 70% specificity [74]. MPM screening using an e-nose was investigated by the same group of researchers, while GC-MS was used in parallel [78]. MLM group was discriminated by control group with a 97% accuracy when GC-MS analyses were processed and with only a 74% accuracy when the data obtained with the e-nose were interrogated. The sensitivity and specificity were at 100 and 91%, respectively, for GC-MS data, and at 82 and 55%, respectively, for e-nose data.

### 5.4. Discrimination between Asthma, COPD and Lung Cancer

**Figure 4:**
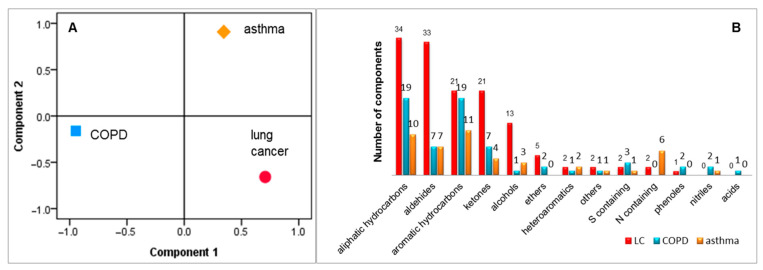
Discrimination between asthma, COPD and lung cancer, based on emitted VOCs (part (**A**)) and classification of collected volatiles according with chemical classes (part (**B**)).

The results discussed above were acquired based on patterns of VOCs or based on reported individual biomarkers. A total number of 146 biomarkers have been reported for all three investigated diseases, as summarised in Table 3. We used the markers collected from the literature to check if they provide discrimination between the investigated diseases. The IBM SPSS statistical software package version 21 was employed for running PCA. Consequently, PCA revealed discrimination between asthma, COPD and lung cancer with 93.3% of variance when the first two principal components were considered, as presented in Figure 4A. The classification of VOCs according with chemical classes is presented in Figure 4B. It is important to mention that the number of lung cancer markers were considerably higher due to the larger number of lung cancer studies.

## 6. The Origin of VOCs Related to the Investigated Diseases and a Sum Up of the Diagnostic Prediction Using the Markers Reported in the Reviewed Studies

A breath sample is composed of a mixture of N_2_, O_2_, CO_2_ and vapors of H_2_O, together with a small fraction of VOCs that consists of more than 1000 compounds [24]. In terms of their origin, these VOCs can be endogenous (generated by the organism, as a normal process of metabolism or as a response to diverse pathologies) or exogenous (absorbed by the organism from the environment and then eliminated through exhaled breath), or both. Unfortunately, the metabolic pathways for the production of endogenous biomarkers associated with various diseases are mostly unknown. The metabolic fates for a limited number of exogenous compounds is well known. The challenge in VOC selection from a complex exhaled breath matrix is the correct identification to a given disease, and this needs to be based on an in-depth knowledge of inflammatory processes. Asthma, COPD and lung cancer are conditions characterized by chronic inflammation and oxidative stress that can be diagnosed through endogenous volatiles. Clinical studies have proven the link between the condition and inflammatory or peroxidative activity as a result of reactive oxygen species (ROS) reaction with lipid membranes [124]. Unfortunately, the inflammatory processes have different sources. For example, sputum inflammatory profiles were able to predict both neutrophilic and eosinophilic asthma [47]. Another method that can be used for asthma phenotyping is sputum cell count [125,126]. However, other interactions of leukocytes, epithelial and stromal cells, proved their contribution to inflammatory processes in asthmatic patients [127].

Hydrocarbons are stable end products of lipid peroxidation released in breath in real time (seconds) after formation in tissues [23]. The presence of alkanes (ethane and pentane) in exhaled breath has been shown to be correlated with lipid peroxidation [24]. However, pentane is also a non-specific marker reported in bowel disease [128] and rheumatoid arthritis [129].

Aldehydes are also associated with oxidative stress and inflammatory processes [4]. Hexanal, heptanal and nonanal, which are formed by the peroxidation of ω 3 and ω 6 fatty acids [59], have been reported as markers of asthma, lung cancer and COPD [36,56,75,76,78,79]. Aldehyde concentrations are known to be affected by age (e.g., pentane may indicate higher metabolic demands of young adults) and smoking history [4]. Endogenous compounds occurring in cigarette smoke (such as acetonitrile, furan, 2-methylfuran, 3-methylfuran, 2,5-dimethylfuran, benzene and toluene) are detected in smokers’ breath samples, but not in the breath of non- or ex-smokers [22,84]. Toluene present in breath samples can result from environmental contamination.

In a pilot study, Gahleitner et al. [65] identified VOC markers of childhood asthma in exhaled breath. Partial least square discriminant analysis was performed and eight compounds (1,7-dimethylnapthalene; 1-(methylsulfanyl)propane; 2-octenal; octadecyne; 1-isopropyl-3-methylbenzene; ethyl benzene; 1,4-dichlorobenzene and limonene) were found to have the greatest contribution to the discrimination between asthmatic and control group. The authors concluded that only 2-octenal is an endogenous marker, while the other seven compounds may potentially result from environmental exposure, catabolism/metabolism, treatments involved for asthma or they can even have an etiological significance in relation to asthma pathogenesis [65].

The concentrations of methanol, acetone, propanol and pentane were measured in patients with lung cancer [69]. The detected concentrations were higher in patients with stage IV than in those with stage III, and in both cases higher in patients with diabetes, than in non-diabetic persons. It was assumed that these findings occurred because the predictive power of markers is proportional with the tumor size and because the lack of insulin is leading into the accumulation of ketones (especially acetone). Patients with smoking history presented increased concentrations of all four markers when compared to non-smokers [69]. In comparison, Song et al. stated that they could not correlate the detected markers (1-butanol and acetoin) with the stage of lung cancer [60].

Isoprene (2-methyl-1,3-butadiene) is an endogenous controversial marker of diseases. The assumption that isoprene is related to cholesterol metabolism [130], is a possible indicator of obesity [131], or a biomarker of lung cancer [16,22,41] and/or COPD [14,54], has been invalidated by researchers. It has been proposed that the variability in isoprene concentration is more related to increasing and decreasing heart rates (as a result of wash-out from muscle tissues [132]) since isoprene concentrations have been shown to increase within a few seconds following physical exercise and then to reach the initial level when breath rate stabilizes [131,133]. Moreover, isoprene can correlate with age, while it was proven that people younger than 40 years exhaled significantly less isoprene than older people [48].

Propanal and 1-propanol have both been proposed as markers of lung cancer [16,22,44,59,69]. However, they are used in disinfectants, and hence are found in high concentrations in the hospitals’ environment. This is why it has been strongly recommended that they are excluded as biomarkers of lung cancer [48]. Benzaldehyde, reported initially as a marker of COPD [14], was actually found to be a decomposition product [19].

Limonene (4-isopropenyl-1-methylcyclohexene) is a ubiquitous monoterpene found in fruits (especially citrus), drinks, flavor additives, air fresheners, cleaning products, scented candles, toothpastes, and deodorants. Therefore, limonene can have possible origin in indoor pollution. Yet limonene has been reported to be an endogenous biomarker of lung cancer [41,78]. This is almost certainly incorrect, and the higher levels in the breath of patients with lung cancer may indicate a higher consumption of citrus fruits or fresh juice [134]. In case of liver cirrhosis, limonene is a key exogenous biomarker denoting a deficient liver metabolism, accumulated due to the liver incapacity to convert it in carveol metabolites or perillyl metabolites by CYP2C enzymes [135,136].

## 7. Limitations, Excluding Criteria and Standardization

The diagnosis of lung disease via breath samples is still not a reality. This is a result of a number of limitations and challenges, including sampling, analysis, confounding factors, correct use of controls, small numbers of volunteers, dietary issues, medications, medical treatments, coexisting conditions, and the lack of reproducibility between studies.

Concerning sampling, it is well documented that subjects are breathing spontaneously with different frequencies, while hypo- or hyper-activity during sampling will produce changes in the composition of the expired breath. Use of mixed expired or end tidal will result in changes in concentrations being measured. Concentrations in the exhaled breath dramatically increase in the end-tidal phase, correlating to the highest concentration of expired carbon dioxide (end-tidal carbon dioxide concentration) [23]. Consequently, the standardization should start from this level. The resting period before sampling and the establishment of which part of breath is going to be sampled need to be decided. In terms of analyses, a combination of GC-MS instrumentation to be used for discovery, followed by fast identification of these targets with rapid techniques such as sensors and e-noses is highly desirable.

The control cohorts used in some studies are often younger compared with the investigated patient groups. For example, in one study, the mean age of the control group was 28 ± 6.08, while the age of the two patient investigated groups, one with COPD with acute exacerbation and the other with COPD only, was 66.9 ± 9.05 and 71.4 ± 7.46, respectively [81]. Fens et al. [38] included in their study a much wider age range, between 18 and 87 years, in an attempt to discriminate between asthma and COPD patients, while Oguma et al. involved 37 volunteers between the ages of 24 and 64 years as controls, and 116 patients with lung cancer between the ages of 36 and 96 years [80]. Comparable age discrepancies were found in another study, where the age difference of the two control groups and the investigated cohort diagnosed with COPD were considerably lower [18]. The mean age of healthy smoker and non-smoker groups were 38.7 ± 14 years, and 42.5 ± 8.4 years, respectively, while the mean age of patients diagnosed with COPD was 56.2 ± 8.5 years. The authors reported as well that the age difference was statistically significant between the two control groups and the group affected by COPD [18]. Conversely, another study included COPD patients with the mean age 58.6 ± 6.9 years, while the mean age of healthy controls was 58.1 ± 8.1 years [20]. Nevertheless, it is still questionable how much the variables such as age, smoking status, Body-Mass-Index, and presence of other diseases can affect the emitted VOCs profiles in an exhaled breath sample. We do believe that a rigorous quantification of emitted volatiles is almost impossible, due to differences in patients, mainly related to gender and age. Adult males with bigger chest volume will definitely exhale more breath compared with females, elders or infants. Whether the concentration of volatile markers of interest is influenced by the total volume, still remains debatable.

The small cohort size involved in many studies is a limitation that needs to be mentioned. Many of the clinical studies included only a few dozen volunteers [13,20,46,57,58,62], and rarely more than two hundred patients [14,22,43,68,84]. In only three cases did the number of included subjects exceeded 400 [73,82,86]. This is understandable, because of the unavailability of suitable patients to donate the necessary samples, but also because of the long duration required to collect a large number of samples. From our personal experience, from a small city with 202,074 inhabitants reported in 2018, we succeeded to collect during one year just 30 tissues samples coming from patients with post-operative bacterial infections and controls [10]. We are confident that other researchers experienced the same issue. For example, Fens et al. [38] mentioned in their published article that although they included 100 patients with an established diagnosis of asthma or COPD, these were recruited over a long period, namely between August 2007 and March 2010. Moreover, the patients come from a limited location. The analyses of these kind of samples may simply provide results that reflect the diagnosis of a subtype/phenotype of a respiratory disease, which cannot accurately be mirrored in the markers liberated by the general population affected by the same condition. For example, Gaida et al. [19] recruited 222 subjects from two different sites in Germany, Hannover and Marburg, in an attempt to investigate VOCs related to COPD. Differences between both room air VOCs and breath VOCs were found when the two sites were compared. Geographical variation in the exhaled VOCs was also found between two sampling sites in China and Latvia [137].

Dietary issues are another important factor connected with VOCs analysis detected from a breath samples. Many studies imposed fasting limits of one hour [40,77,95], two hours [15,38,47,63], three hours [65,81], four hours, [14] and six hours [33]. In some studies, volunteers were fasting overnight, or for 12 hours [21,41,60,70,82]. However, a long fasting period is not easily accepted by volunteers, and is not feasible in a real-life scenario.

The impact of medication applied for respiratory diseases (like inhalative agents, corticosteroids, antibiotics, anesthetics, etc.) together with the effect of concomitant medications (antihypertensive or anti-diabetic therapy), as well as the effect of co-existing disorders on exhaled VOCs still remains unknown.

Owing to a total lack of standardization in this field, different excluding criteria have been applied. For example, Zou et al. [70] excluded all participants younger than 45 years old from a validation cohort, while Phillips et al. [14] excluded all patients with current or previous cancer history, known dementia, heart failure, other known pulmonary, and renal or liver disease when investigating COPD. Rodríguez-Aguilar and colleagues in their COPD study excluded all patients with asthma and all individuals with a history of upper or lower respiratory tract infection during the 4 weeks before their measurements [85]. Van Vliet investigated asthma in children aged between 6 and 17 years old, and applied the following exclusion criteria: technically unsatisfactory performance of lung function measurements; other pulmonary diseases; cardiac abnormalities; mental retardation; congenital abnormalities or existence of a syndrome; active smoking; children that had immunotherapy during the study [76].

Excluding certain categories of volunteers is not a suitable solution all of the time. Furthermore, patients often do not honestly declare if they are active or ex-smokers. In addition, their medical histories are often confidential. Applying excluding criteria will decrease the cohort in a biased way. However, not applying such criteria may result in too much interference that makes it difficult to follow the pattern of markers occurrence, which, in turn, will finally affect the diagnostic accuracy. Perhaps the best decision is to not exclude a key population, but just to subtract some well-known volatiles associated to certain habits (e.g., smoking).

The chemical composition of a breath sample is also dependent on the lung area from where it is sampled. Alveolar breath is generally expected to have the highest concentration of VOCs, because it originates from the deepest part of the lungs, and is, therefore, the closest to the alveolar capillaries, but that depends on the solubility of the volatile, which is related to the compound’s Henry coefficient. Clearly, the gas exchange process is dependent on the alveolar membrane thickness and in the case of respiratory disease by the ability of patients to take a deep inspiration and provide a profound expiration. The lack of standardized methods for sampling, analysis and data processing, as well as the effects of environmental contaminants, has resulted in the large number of disparate studies.

An important issue to address is where in the breathing cycle a breath sample should be taken from patients suffering from COPD or asthma, because these illnesses are more related to the upper airways, and not the alveolar region. Whilst it is true that breath from the lower airways are less important for these diseases, use of the end-tidal region limits dilution and contamination of a breath sample from the mouth, and anatomic or tubing dead-space. Furthermore, there would no temporal resolution in the breath sample that could be used to differentiate upper from lower airways breath samples. Therefore, it is always best to collect a breath sample during the end-tidal exhalation phase.

### 7.1. Current Status of VOCs Based Diagnosis

**Figure 5:**
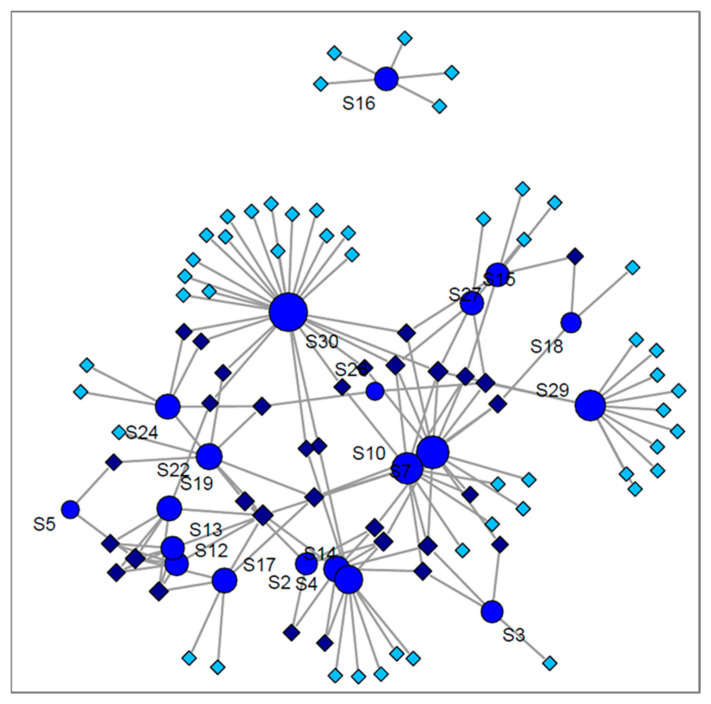
Network analyses of lung cancer studies distribution based on detected volatiles, where circles marked with S represent the number of the study, allotted similarly in Table 3; darker diamonds represent the common VOCs; pale diamonds depict the uncommon VOCs.

A snapshot of cancer studies included in the current review, including quantification or identification, is presented in Figure 5, as a network analyses obtained by using R studio with console version 3.6.3. It is worth mentioning that in 21 studies related to lung cancer, 83 biomarkers have been reported. From this number, just 31 of them are common between at least two studies. Moreover, the best concordance was obtained for 2-butanone, which was common between six studies, followed by different isomers of xylene detected in five studies. Nonanal, 2-pentanone, 3-hydroxy-2-butanone and hexanal were common markers in four studies.

**Figure 6:**
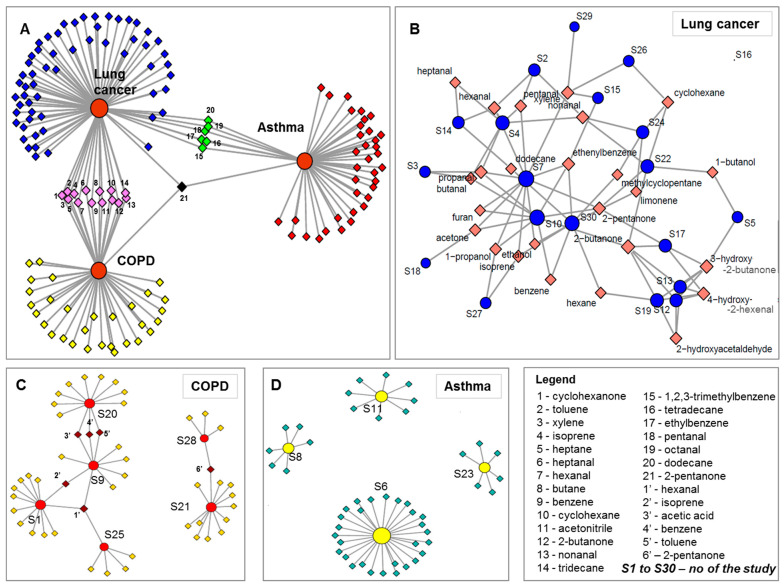
Network analyses presenting the distribution of VOCs between the three reviewed conditions (part (**A**)) and highlighting volatiles common dispensation in case of lung cancer (part (**B**)), COPD (part (**C**)) and asthma (part (**C**)). The circles noted with S (in part (**B**–**D**)) represent number of the study, which are allocated similarly in Table 3, and the diamonds represent the components.

The case of the other two lung diseases is even more deficient in comparison to lung cancer. No common compound was found for asthma, for which four studies only reported biomarker identification. Six studies reported biomarker identification for COPD. Just one compound, hexanal, was common between three studies, while five VOCs were common only in two studies. The distribution of VOCs between the three diseases we have reviewed, as well as between different studies investigating the same conditions are presented in Figure 6. As shown in Figure 6A, the compounds that are common between lung cancer studies and COPD studies are generally common for all three diseases. This fact denotes that they are not specific markers for a given lung condition, but rather simply indicative of a respiratory disease. Consequently, it is obvious that exhaled VOCs may depend also on a variety of parameters, other than the disease under investigation. This is why a standardized approach, including simultaneously sampling, analysis, data processing, normalization and correcting parameters, is needed to lead to the discovery of well-founded biomarkers that can provide clinically relevant information from breath analysis. Janssens et al. [138] have reviewed VOCs detected from urine, tissue, blood and cell lines of lung cancer patients and discovered some similar markers with those reported in the present review.

Efforts for development of a new standardized sampling device are being made by the company Owlstone Medical (Cambridge, UK). Their ReCIVA (Respiration Collector for In Vitro Analysis) provides a dedicated clean air supply, CASPER (Clean Air Supply Pump for ReCIVA). Thermo-desorption tubes containing Tenax/Carbograph-5TD adsorbents are used to collect the breath samples. The ReCIVA device allows for specific fractions of exhaled breath to be collected in TD tubes through continuous monitoring of pressure and CO_2_ levels within the mask and for the removal of background contaminants [139]. Using Tenax as an absorption material in the sampling process has advantages (such as stability and low desorption temperature) but there are some drawbacks. For example, benzaldehyde is a decomposition product that appears in the chromatograms when Tenax tubes are used. In addition, nonanal and decanal, which have been proposed as markers related to COPD [79], asthma [76] and lung cancer [56,59,78], are difficult to evaluate correctly when Tenax^®^ TA is used as adsorption material [19].

### 7.2. Overall Proposed Solutions

- Breath sampling needs highly standardized conditions to include certain breath fraction, well-defined excluding criteria, given conditions for preparation of volunteers for sample collection, and the volume and duration of sampling;
- In the absence of a “perfect” breath reference material, routine breath control measurements should be performed at certain time spans;
- Operating of instruments according to well-defined protocols and standardized criteria;
- Monitoring of background air that can impact the performance of the methods;
- Calibration of instruments (especially sensors) with standardized samples that mimic breath is highly desired;
- Data processing workflow should be also standardized including for examples: peaks alignment, normalization, and statistical analyses.
- Utilization of standardized methods for data processing (statistical tools, thresholds used for extraction parameters);
- Creation of databases of markers obtained using standardized methods that can be accessed and completed by researchers.

## 8. Concluding Remarks and Future Perspectives

The current available tools for the diagnosis of pulmonary diseases based on exhaled VOCs are promising, but are far from being of clinical use. Promising findings have been reported, and we have emphasized in this review that both discrimination between the three lung diseases reviewed and diagnosis prediction are relevant. However, multiple constraints—including sampling, analysis, validation and standardization—need to be solved before analysis of specific VOCs can be widely applied into clinical practice. As a short-term future perspective, we predict that analytical instrumentation will be used in small point of care studies to confirm or deny the possibility of certain respiratory conditions. Based on this first diagnosis the subjects may then be sent for a more complex and confirmatory diagnosis. As for long-term future perspectives, we consider that online instrumentation, especially portable instrumentation, IMS, GC-IMS, sensors and e-noses, are convenient devices for physicians to be used in the diagnosis and monitoring of respiratory diseases, as well as for use in monitoring therapy.

## Figures and Tables

**Table 1:** General view of included clinical studies in terms of: approach, number of subjects and framework.

Approach/ Comparison	Matrix/Sample	Cohort Size *	Patients (Smokers/Former/ Never)	Controls (Smokers/Former/ Never)	Organization	Reference
COPD vs. controls	mixed breath	66 + 45	40/20/6	12/10/23	University Hospital Maastricht; Centre for Integrated Rehabilitation Organ Failure, Horn, The Netherlands	[54]
Asthma vs. controls (children)	mixed breath	63 + 57	na	na	Department of Pediatric Pulmonology of University Hospital, Maastricht, Netherlands	[17]
Asthma vs. controls	mixed + alveolar breath	27 + 24	all non-smokers	all non-smokers	Istituto Dermopatico dell’ Immacolata, Rome (Italy)	[55]
Lung cancer vs. controls	mixed breath	28 + 36	0/17/11	36 ^	Forlanini Hospital, Roma, Italy.	[21]
Lung cancer vs. never vs. smoker vs. controls	alveolar breath	12 + 12 + 12	0/12/0	12//0/12	Department of pulmonology, University Rostock, Rostock, Germany	[56]
COPD vs. controls	alveolar breath	20 + 6	18/2 ^	0/6 ^	Medicines Evaluation Unit, Wythenshawe Hospital, Manchester, UK	[57]
Lung cancer vs. never vs. smoker vs. controls	alveolar breath	31 + 31 + 31	0/29/2	31/0/31	Department of pneumology of local hospitals from Rostock, Germany and Innsbruck, Austria	[48]
Lung cancer vs. controls	alveolar breath	30 + 22	not specified	not specified	Oncology Division, Rambam Health Care Campus, Haifa, Israel	[58]
Lung cancer vs. non-smokers	mixed breath	40 + 38	21/12/7	0/10/28	Thoracic Surgery Section of the University Hospital of Parma, Italy	[59]
Lung cancer vs. controls	mixed breath	43 + 41	0/21/22	0/0/41	Affiliated Hospital of Anhui Medical University, Hefei, Anhui, China.	[60]
Asthma vs. control (children)	mixed breath	35 + 15	na	na	Department of Paediatric Immunoalergology of Hospital D. Pedro, Aveiro, Portugal	[61]
Asthma vs. controls	mixed breath + sputum	35 + 23	0/1/34	0/0/23	Medicines Evaluation Unit, Wythenshawe Hospital, Manchester, UK	[47]
Emphysema/COPD vs. controls	alveolar breath	43 + 161	ns	ns	Institute for Molecules and Materials, Radboud University, Nijmegen, the Netherlands	[32]
Asthma vs. COPD	mixed breath	60 + 40	No healthy controls	5/51/4 COPD:13/0/27	Academic MC Amsterdam; Haga Teaching Hospital; Albert Schweitzer Hospital, Dordrecht, the Netherlands	[38]
COPD with AATD vs. COPD vs. controls	exhaled breath condensate	10 + 23 + 10	3/30/0	0/2/8	Philipps University Marburg; Ludwig Maximilians University Munich; Saarland University Hospital, Homburg/Saar, Germany	[39]
Lung cancer vs. controls	mixed breath	23 + 30	2/0/21	6/0/24	Department of Lung Disease, Collegium Medicum, Nicolaus Copernicus University, Torun, Poland	[62]
Lung cancer vs. controls	alveolar breath	137 + 143	ns/ns/ns	102/ns/41	Department of Lung Disease, Collegium Medicum, Nicolaus Copernicus University, Torun, Poland	[22]
Asthma vs. controls (children)	mixed breath	32 + 27	na	na	The Hospital Infante D. Pedro E.P.E, Aveiro, Portugal	[46]
COPD vs. controls	breath + sputum	39 + 32	12/27/0	10/0/22	Medicines Evaluation Unit, University Hospital of South Manchester, UK.	[63]
COPD vs. COPD with BC vs. controls	alveolar breath	30 + 54 + 35	ns/ns/ns	ns/ns/ns	KIST Europe; Max Planck Institute; Cluster of Excellence for Multimodel Computing and Interaction; Saarland University, Germany	[35]
COPD vs. controls	alveolar breath	119 + 63	41/78/0	6/18/39	Respiratory Unit, Prince Philip Hospital, Llanelli, UK	[14]
COPD vs. asthma vs. controls	exhaled breath condensate	17 + 20 + 7	5/16/16	0/3/4	Patients recruited from hospital out-patient clinics; controls from the community in Sydney, Australia.	[40]
Lung cancer vs. controls	alveolar breath	29 + 44	ns/ns/ns	ns/ns/ns	Department of Lung Disease, Collegium Medicum, Nicolaus Copernicus University, Torun, Poland	[44]
Lung cancer vs. controls	mixed breath	92 + 137	25/58/9	28/71/35	Outpatient clinic from Cleveland Clinic, Cleveland, Ohio	[64]
Asthma vs. controls (children)	alveolar breath	11 + 12	na	na	Inaccessible for authors; study developed in UK	[65]
Asthma with exacerbation vs. asthma (children)	mixed breath	16 + 26	na	na	Outpatient clinic, Department of Pediatric Pulmonology, Maastricht University Medical Centre, Maastricht, the Netherlands	[45]
Lung cancer vs. controls	mixed breath	22 + 10	19 ^#^/3	0/10 ^	Local hospitals from Linköping, Sweden.	[66]
Lung cancer vs. BPD vs. controls	mixed breath	97 + 32 + 88	ns/ns/ns	45/43 ^	James Graham Brown Cancer Center, University of Louisville, Louisville, Kentucky	[67]
Asthma vs. controls	mixed breath	195 + 40	ns/ns/ns	ns/ns/ns	High Altitude Clinic, Davos-Wolfgang, Switzerland	[43]
Lung cancer vs. BPD vs. controls	mixed breath	107 + 40 + 88	56/65/12	45/0/43	Unmentioned; study developed in Louisville, Kentucky	[68]
Lung cancer vs. controls	mixed breath	50 + 39	33 ^#^/17	7 ^#^/32	St. Marianna University School of Medicine, Kanagawa, Japan	[36]
Lung cancer vs. controls	mixed breath	13 + 25	ns/ns/ns	ns/ns/ns	Shanghai Chest Hospital, Shanghai, China	[69]
Lung cancer vs. BPD vs. controls	alveolar breath	79 + 54 + 38	15/40/24	9/20/9 12/25/17 (BPD)	Sir Run Run Shaw Hospital, Hangzhou, China	[70]
COPD vs. smoker vs. non-smoker controls	alveolar breath	45 + 23 + 28	5/40/0	11/12/28	Department of Pneumology, Ruhrlandklinik, University Hospital of Essen, Germany	[18]
Lung cancer vs. BPD vs. smoker vs. non-smoker controls	mixed breath	85 + 34 + 45 + 40	45/34/2/4 ^λ^	45/0/40 10/7/7/10 ^λ^ (BPD)	James Graham Brown Cancer Center, University of Louisville, Louisville, Kentucky	[71]
Lung cancer vs. controls	mixed breath	13 + 25	5/8 ^	8/17 ^	Shanghai Chest Hospital, Shanghai, China	[72]
Lung cancer vs. BPD vs. controls	mixed breath	165 + 65 + 194	69/80/7	25/20/20 (BPD) 73/41/80	Unmentioned; study developed in Louisville, Kentucky	[73]
COPD vs. controls	mixed breath	89 + 101	37/52/0	49/52 ^	Local hospitals in Marburg & Hannover, Germany.	[19]
COPD vs. controls	alveolar breath	79 + 73	42/37 ^	41/32 ^	Unmentioned; study developed in Germany	[37]
MPM vs. AEx vs. controls	alveolar breath	23 + 22 + 21	9/5/9 (MPM)	5/5/12 (AEx) 13/0/8	University Hospitals of Ghent, Leuven and Antwerp, Belgium	[74]
Lung cancer vs. controls	mixed breath	21 + 22	4/7/10	9/5/8	Unmentioned; study developed (probably) in Israel	[33]
Lung cancer vs. controls	mixed breath	37 + 23	21/14/2	4/7/12	ELK Berlin Chest Hospital and Charité Universitäts Medizin, Visceral, Vascular and Thoracic Surgery, Berlin, Germany	[75]
Asthma vs. asthma with exacerbations (children)	mixed breath	49 + 45	na	na	Outpatient clinic of 2 specialized pediatric pulmonology centers in the Netherlands	[76]
Lung cancer vs. BPD	alveolar breath	89 + 30	16/56/17	14/10/6	Sheba Medical Center, Tel Hashomer, Israel.	[77]
MPM vs. AEx vs. controls	mixed breath	14 + 19 + 16	1/9/4	6/7/6 (AEx) 0/8/8	Three participating university hospitals from Belgium	[78]
COPD vs. never vs. former vs. smokers	alveolar breath	57+ 33 + 28 + 39	8/49/0	32/28/39	Hospital Central de la Defensa “Gomez Ulla”, Madrid, Spain	[79]
Lung cancer vs. controls	mixed breath	116 + 37	42/51/23	2/5/30	Tokai University Hospital, Kanagawa, Japan	[80]
Lung cancer vs. controls	alveolar breath	107 + 29	47/15/45	5/3/21	Aichi Cancer center, Nagoya Japan	[16]
AECOPD vs. COPD vs. controls	alveolar breath	14 + 16 + 24	6/7/1 (AECOP)	3/13/0 (COPD) 7/0/17	Department of Internal Medicine II, Medical University of Innsbruck, Austria	[81]
COPD vs. controls	exhaled breath condensate	22 + 14	10/11/1	5/8/1	Unmentioned; study developed in Switzerland	[20]
Lung cancer vs. BPD vs. controls	alveolar breath	233+ 111 + 140	102/45/86	41/16/54 (BPD) 69/16/55	Sir Run Run Shaw Hospital, Hangzhou, China	[82]
Lung cancer vs. controls	alveolar breath	37 + 48	9/15/13	4/10/34	Seoul National University Bundang Hospital, Republic of Korea	[15]
Lung cancer vs. controls	mixed breath	57 + 72	22/35 ^	25/47 ^	First Affiliated Hospital of Jinan University, Guangdong, China	[41]
Lung cancer vs. COPD vs. controls	mixed breath	30 + 18 + 61	12/16/2	10/8/0 (COPD) 13/21/27	Hospital Clinic and Hospital Universitari Sagrat Cor of Barcelona, Spain.	[83]
Lung cancer vs. controls	mixed breath	30 +30	19/11 ^	5/25 ^	Chinese People’s Liberation Army General Hospital, Beijing, China	[34]
Lung cancer vs. controls	alveolar breath	108 + 121	69/39 ^	50/71 ^	Department of Lung Disease, Collegium Medicum, Nicolaus Copernicus University, Torun, Poland	[84]
Asthma vs. CF vs. controls (children)	mixed breath	20 + 13 + 22	na	na	Maastricht UMC & Department of Paediatric Respiratory Medicine, Maastricht, Netherlands	[42]
COPD vs. controls	mixed breath	25 + 33	4/10/11	3/7/23	Outpatient clinic in Hospital Central “Dr. Ignacio Morones Prieto”, San Luis Potosi, Mexico	[85]
Lung cancer vs. controls	alveolar breath	462	ns/ns/ns	ns/ns/ns	five medical centers in USA	[86]
Lung cancer vs. controls	mixed breath	15 + 14	3/12/0	1/6/7	University hospital of Liège, Belgium	[13]

* the numbers presented are connected with the categories of diseased patients or controls involved in the study (the first number refers to patients and the second to controls); #—former + active smokers; ^—former + never smokers; λ—smoking status unknown; AECOPD = acute exacerbation chronic obstructive pulmonary disease; AEx = asymptomatic former asbestos; BC = bronchial carcinoma; BPD = benign pulmonary diseases; CF—cystic fibrosis; MPM = malignant pleural mesothelioma (tumor of pleural lining of the thorax associated with asbestos exposure).

**Table 2:** Details of clinical studies reviewed.

First Author/ Year [Ref]	Diseases	Observations and Details of Diseases	Analytical Platform	Outcomes	Statistical Approach/Representation	Accuracy (%) *
Van Berkel, 2010 [54]	COPD	steroid-naïve patients	TD-GC-ToF-MS	1179 VOCs; 12 identified markers	SVMs; John Platt’s SMO; RF	-
Dallinga, 2010 [17]	Asthma	children; atopy; allergy	GC-MS	945 VOCs; 10 discriminative VOCs	DA	92–100
Montuschi, 2010 [55]	Asthma	intermittent & persistent mild; atopy	GC-MS + E-nose	FENO monitoring	*t* test; Mann-Whitney U test; feed-forward neural network; PCA	DP: 70.8–95.8
D’Amico, 2010 [21]	Lung cancer	Adenocarcinoma, SCLC, bronchio-alveolar & squamous cell carcinoma	E-nose + GC-MS	pattern of VOCs	PLS-DA; PLS-LVs	79–86
Fuchs, 2010 [56]	Lung cancer	SCLC & NSCLC	SPME-OFD—GC-MS	10 discriminative aldehydes	Kruskal–Wallis one-way ANOVA; Box plot.	-
Basanta 2010 [57]	COPD	GOLD I to IV, exacerbation, eosinophilia	GC-DMS	VOCs profile	Pearson chi square, students *t*-test, Mann Whitney U, PCA, ROC, DFA, Pearson’s correlation coefficient	76–84 AUC: 79–92
Kischkel, 2010 [48]	Lung cancer	SCLC & NSCLC	GC-MS	42 VOCs, 4 identified markers	Mann–Whitney Rank test; ANOVA; post hoc Student–Newman–Keuls; Dunn’s Method; PCA,	-
Peng, 2010 [58]	Lung cancer	Stage I to IV	GC-MS & GNPs nanosensor array	pattern of VOCs	PCA	-
Poli, 2010 [59]	Lung cancer	Stage I or II; NSCLC	SPME-OFD–GC-MS	7 identified markers (aldehydes)	ANOVA; ANCOVA; Tukey’s post hoc test; DA (Wilks’ Lambda).	AC: 93–97 OCC:92
Song, 2010 [60]	Lung cancer	Stage I to IV; squamous cell, adenocarcinoma	GC-MS	VOCs profile; 2 selected markers	Wilcoxon rank sum test; ROC	AUC: 94–96
Caldeira, 2011 [61]	Asthma	Children; allergic & allergic rhinitis	GC–qMS	44 VOCs; 28 discriminative VOCs	ANOVA; PLS; PLS–DA;	CVA: 88
Ibrahim, 2011 [47]	Asthma	Eosinophilia; neutrophilia	GC-MS	47 discriminative VOCs	MLR, PCA, Box plot; ROC	CVA: 82 AUC: 90–98
Cristescu, 2011 [32]	COPD	GOLD I to III; emphysema	PTR-MS	31 discriminative VOCs	logistic regression, ROC	AUC: 0.56
Fens 2011 [38]	Asthma & COPD	fixed & classic asthma; COPD GOLD stages II & III	E-nose	pattern of VOCs	CDA; PCA, ROC	AUC: 93–95
Hattesohl 2011 [39]	COPD	with & without alpha 1-antitrypsin deficiency	E-nose	pattern of VOCs	LDA, Mahalanobis distance, Mann–Whitney U-test, Wilcoxon signed rank	CVA: 58.5–80.5
Rudnicka 2011 [62]	Lung cancer	SCLC, squamous cell, adenocarcinoma	GC–TOF/MS	55 VOCs	Mann–Whitney U test DA, FA	-
Ulanowska, 2011 [22]	Lung cancer	Adenocarcinoma; Planoepitheliale.	GC/MS	VOCs profile; 14 identified markers	DA, FA, CHAID tree	-
Caldeira, 2012 [46]	Asthma	Children; allergic & allergic rhinitis	GC×GC–ToF-MS	134 VOCs; 6 identified markers	PLS-DA, MCCV	-
Basanta 2012 [63]	COPD	GOLD I to IV	GC-ToF-MS	487 VOCs 11 discriminative VOCs	Mann Whitney U, DFA, LOOCV, PCA, ROC	69; AUC:74–95
Hauschild 2012 [35]	COPD & BC	ns	MCC/IMS	VOCs profile; 20 discriminative VOCs	Decision tree, linear SVM, naive Bayes, neural net, radial SVM, RF	82–94; AUC: 80–92
Phillips, 2012 [14]	COPD	GOLD I to IV, emphysema	GC-MS	VOCs profile	12 automatic classifier methods; with 8 stand-alone classifiers, and 2 ensemble techniques	71–82; AUC: 71–82
Timms 2012 [40]	Asthma & COPD	both with & without gastro-oesophageal reflux	E-nose	pattern of VOCs	PCA, Mahalanobis distance, Mann–Whitney, Kruskal–Wallis & t tests	65–85
Buszewski, 2012 [44]	Lung cancer	SCLC & NSCLC	GC-ToF-MS & canine recognition	VOCs profile	Kruskal–Wallis test, chi^2^ test, factor analysis, PCA	-
Mazzone, 2012 [64]	Lung cancer	Stage I to IV; SCLC, NSCLC, squamous cell, adenocarcinoma	colorimetric sensor array	bio-signatures of lung cancer	ROC, *t*-tests, Pearson test, four logistic prediction models	46–89; AUC:81–85
Gahleitner, 2013 [65]	Asthma	Children;	GC-MS	VOCs profile; 8 identified markers;	PLS-DA, PCA, Whisker box plots, two-tailed *t*-test,	-
Robroeks, 2013 [45]	Asthma	Children; atopy, exacerbations	GC-TOF-MS	VOCs profile; 6 discriminative VOCs	SMV, *t*-test, Friedman test	64–100
Schmekel, 2014 [66]	Lung cancer	Stage III & IV; SCLC, NSCLC, adenocarcinoma, squamous cell	E-nose	pattern of VOCs	AAN, PLS	CVA:98
Fu, 2014 [67]	Lung cancer	Stage I to IV; SCLC, NSCLC, adenocarcinoma, squamous cell	FT-ICR-MS	Detection of C_1_ to C_12_ carbonyls; 4 identified markers	Wilcoxon test, box plot	-
Meyer, 2014 [43]	Asthma	asthma endotypes & phenotypes	GC-MS	945 detected VOCs; 16 discriminative VOCs	cluster analyses	83–95; DP: 98–99
Bousamra, 2014 [68]	Lung cancer	BPD & cancer stage 0, I, II	FT-ICR-MS	Detection of carbonyls; 4 identified markers	Wilcoxon test, box plot	-
Handa, 2014 [36]	Lung cancer	Stage I to IV; squamous cell, adenocarcinoma, SCLC	MCC-IMS	115 VOCs, 9 identified markers	Box-and-Whisker plots, Wilcoxon-Mann-Whitney test,	-
Ma, 2014 [69]	Lung cancer	Stage III & IV	GC×GC-FID	quantification of benzene series; 5 identified markers	Mann–Whitney U Test, Wilcoxon W test, PLS-DA	-
Zou, 2014 [70]	Lung cancer	Stage I to IV; SCLC, NSLC, adenocarcinoma, squamous cell, Adeno-squamous carcinoma	GC-MS	5 identified markers	Pearson’s χ2 test, Mann–Whitney test, ROC, PCA	AUC: 0.67–0.88
Besa, 2015 [18]	COPD	GOLD I to IV	MCC/IMS	224 VOCs; 6 discriminative VOCs	Kolmogorov–Smirnov test, one way ANOVA, Kruskal–Wallis test, *t*-test, Mann–Whitney U-tests	67–71
Li, 2015 [71]	Lung cancer	SCLC, NSLC, squamous cell, adenocarcinoma,	FT-ICR-MS	Detection of C_1_ to C_10_ carbonyls; 6 identified markers	Kruskal–Wallis test, boxplot, PLS, SVM, RF, LDA, ROC	89–97
Ma, 2015 [72]	Lung cancer	ns	TD-GC-MS;	5 identified markers	quantification with standards	-
Schumer, 2015 [73]	Lung cancer	Stage I to IV; SCLC, NSLC, squamous cell, carcinoid, large cell, adenocarcinoma in situ, adenocarcinoma	FT-ICR-MS	4 identified markers (carbonyls)	Not specified	69
Gaida, 2016 [19]	COPD	GOLD III & IV	GC-MS	134 VOCs; 14 identified markers	*t*-test, ANOVA, Newman–Keuls test, LDA,	CVA: 78–86
Allers, 2016 [37]	COPD	Not specified	GC-IMS & GC-APCI-MS	45 VOCs (by GC-IMS) & 102 (by GC-APCI-MS);	Welch’s *t*-test, Welch’s *t*-test, Box-and-whisker plots	-
Lamote, 2016 [74]	Lung cancer	MPM; AEx	MCC-IMS	pattern of VOCs	Fisher’s exac*t*-test, Kruskall–Wallis test, One-way ANOVA, ROC, LOOCV	76–87; AUC:17–94
Feinberg, 2016 [33]	Lung cancer	Naïve LC patients; Stage III & IV; SCLC, squamous cell, adenocarcinoma	PTR-MS	pattern of VOCs	paired two-tailed Student *t*-test, box plot	-
Schallschmidt 2016 [75]	Lung cancer	Not specified	GC-MS	24 quantified VOCs; 7 markers	Mann–Whitney U-test, LDA, LOOCV, Box plot, Cluster analysis,	-
van Vliet, 2017 [76]	Asthma	Children, exacerbation, atopy	GC-ToF-MS	7 identified markers	RF, ROC, PCA	OCC: 82 AUC: 90
Shlomi, 2017 [77]	Lung cancer	Stage: I to IV;	E-nose	pattern of VOCs	DFA, LOOCV, Wilcoxon test, ANOVA, chi-square test	76-87
Lamote, 2017 [78]	Lung cancer	Naïve MPM, pleural plaques	GC-MS, E-nose	VOCs profile; 6 identified markers;	DA, ROC	50–97; AUC: 36–98
Jareno-Esteban, 2017 [79]	COPD	Not specified	GC-MS	5 VOCs with discriminative features	Kolmogorov–Smirnov test	-
Oguma, 2017 [80]	Lung cancer	Stage: I to IV; SCLC, NSLC, squamous cell, adenocarcinoma	GC-MS	Quantification of 14 pre-stabilized VOCs; 2 markers	ROC, Mann–Whitney U-test, Kruskal–Wallis test, Wilcoxon’s rank test, Jonckheere–Terpstra test	AUC: 67–71
Sakumura, 2017 [16]	Lung cancer	Stage: I to IV	GC-MS	63 VOCs, 20 discriminative VOCs	Wilcoxon test, Kolmogorov–Smirnov test, SVM, LOOCV	AC: 89; DP: 84.6
Pizzini, 2018 [81]	COPD	GOLD: II, III & IV; AECOPD, stable COPD	GC-MS	105 VOCs; 12 identified markers	One way ANOVA, post hoc analysis, RF	AUC: 92–97
Bregy, 2018 [20]	COPD	GOLD: I to IV	SESI-HRMS	1441 detected VOCs; 43 discriminative VOCs	LOOCV, PCA, Venn diagram, ROC	89
Wang, 2018 [82]	Lung cancer	Not specified	TD-GC-MS SPME-GC-MS	pattern of VOCs; 12 identified markers	chi-square, ROC	77–83 AUC: 73–88
Chang, 2018 [15]	Lung cancer	Stage: I to IV; squamous cell; adenocarcinoma	seven metal oxide gas sensors	pattern of VOCs	SVM, LDA, PCA	61–75
Cai, 2018 [41]	Lung cancer	SCLC & NSLC	E-nose	pattern of VOCs	Mann-Whitney U test, chi-square test, ANOVA, ROC,	76/94/83
Guirao, 2019 [83]	Lung cancer & COPD	Stage: I & II; SCLC, NSLC, lepidic adenocarcinoma, adenocarcinoma, squamous cell	Trained dogs	Pattern recognition	ROC	AUC: 0.98
Sun, 2019 [34]	Lung cancer	squamous cell, adenocarcinoma	PTR-MS	5 discriminative VOCs	Mann–Whitney U test, LDA, whisker box plot, ROC	AUC: 0.74–0.99
Rudnicka, 2019 [84]	Lung cancer	Stage: I to IV; SCLC, NSLC	GC–TOF/MS	86 VOCs;	Mann–Whitney U test, DFA, FA, AAN, BOX PLOT, ROC	AUC: 0.86
Bannier, 2019 [42]	asthma	moderate to severe asthma & children with CF	E-nose	pattern of VOCs	AAN, ROC, LOOCV,	AUC: 0.79–90
Rodríguez-Aguilar 2019 [85]	COPD	GOLD: I to IV	FCG E-nose	62 VOCs; 17 discriminative VOCs;	Student test t, Mann–Whitney U-test, Fisher test, SPCA, ROC, LOOCV	OCC: 82;
Phillips, 2019 [86]	Lung cancer	Not specified	GC-MS	pattern of VOCs	ROC	80-88; AUC: 77–88;
Pesesse, 2019 [13]	Lung cancer	Not specified	TD-GC-GC×GC-ToF-MS	37 VOCs with discriminative features	Fisher test, PCA, RF	-

*—or stated otherwise; na—not applicable; AAN—artificial neural nets; AATD—1-antitrypsin deficiency; AEx—asymptomatic former asbestos; AUC—The area under the ROC; BC—bronchial carcinoma; BPD—benign pulmonary diseases; CDA—canonical discriminant analysis; CVA—Cross-Validated Accuracy-Value; DA—Discriminant analysis; DFA—Discriminant function analysis; DP—diagnostic performance; FA—factor analysis; FCG e-Nose—ultrafast gas-chromatography equipped with electronic nose detector; FENO—fractional exhaled nitric oxide; FT-ICR—Fourier transform-ion cyclotron resonance; GNPs—gold nanoparticles; GOLD—Global Initiative for Chronic Obstructive Lung Disease; LDA—Linear discriminant analysis; LOOCV—leave-one-out cross-validation; LPPI-MS—low pressure photoionization mass spectrometry; MCCV—Monte Carlo Cross Validation; MLR—multivariate logistic regression, MPM—Malignant pleural mesothelioma; ns—not specified; NSCLC—non-small-cell lung carcinoma; OCP—overall correct classification; PCA—principal component analysis; PLS-D—Partial Least Squares Discriminant analysis; PLS-LVs—Partial Least Squares Latent Variables; RF—random forest; ROC—Receiver Operating Characteristic Curve; SCLC—small-cell lung carcinoma; SESI-HRMS—secondary electrospray ionization—high-resolution mass spectrometry; SMO—sequential minimal optimization algorithm; SPME-OFD–GC-MS—Solid phase microextraction on-fiber derivatization; SVM—Support vector machine.

**Table 3:** Markers associated with the three reviewed respiratory diseases.

No	First Author/ Reference	Marker of	Detected Markers	Concentration in Patients *			Concentration in Controls *			Unit	*p*-Value
				Smokers	Former	Never	Smokers	Former	Never		
1	Van Berkel, [54]	COPD	12 VOCs (nq)	2,4,6-trimethyl-decane; 2,6-dimethyl-heptane; 3,7-dimethyl 1,3,6 octatriene; 4,7-dimethyl-undecane; 4-methyl-octane; benzonitrile; hexadecane; hexanal; isoprene; octadecane; terpineol; undecane							
2	Fuchs, [56]	LC	hexanal	-	1.632	-	0	-	0.172	ng/L	-
			nonanal	-	1.768	-	0	-	0		-
			octanal	-	6.652	-	0.768	-	1.407		-
			pentanal	-	33.957	-	12.077	-	4.689		-
3	Kischkel, [48]	LC	dimethyl sulphide	-	0.27	-	0.27	-	0.3	nmol/L	=0.002
			dimethyl formamide	-	5589.5	-	1403	-	558.5	counts	=0.003
			propanal	-	0.34	-	0	-	0	nmol/L	<0.001
			butanal	-	6.47	-	1.06	-	1.41	nmol/L	<0.001
4	Poli, [59]	LC	propanal	-	49.8	66.3	-	-	24.4	pM^1^	=0.006
			butanal	-	23.6	28.6	-	-	10.8		<0.001
			pentanal	-	17.1	20.3	-	-	8.2		<0.001
			hexanal	-	38.2	35.9	-	-	10.3		<0.001
			heptanal	-	15.4	17.0	-	-	6.9		<0.001
			octanal	-	26.9	22.4	-	-	11.6		<0.001
			nonanal	-	51.7	36.5	-	-	13.3		<0.001
5	Song, [60]	LC	1-butanol	2.21–30.31	-	-	-	-	0.32–13.97	ng/L	<0.005
			3-hydroxy-2-butanone	1.95–50.3	-	-	-	-	>6.21		<0.005
6	Cristescu, [32]	asthma	30 proposed VOCs (nq)	1-(3-pyridinyl)-ethanone; 1-(4-pyridinyl)-ethanone; 1,2,3,5-tetramethylbenzene; 3-pentanone; 1,2,3-trimethylbenzene; 1,2,4,5-tetramethylbenzene; 1,2,-diethylbenzene; 1,3,-diethylbenzene; 1,4,-diethylbenzene; 2-(1-methylethyl)-pyridine; 2,3-butanedione; 2,6-dimethyl-benzenamine; 2-methyl-3-buten-2-ol; 2-methylbutanal; 2-pentanone; 2-propyl-pyridine; 3-methyl-2-butanone; 3-methyl-2-buten-1-ol; 3-methyl-3-buten-1-ol; sec-butylbenzene; pentanal; 3-methylbutanal; 4-aminobenzenecarbonal; benzamide; benzeneethanamine; chloramine; N,N-dimethyl-benzenamine; butylbenzene; n-ethyl-benzenamine; propiolonitrile;							
7	Ulanowska, [22]	LC ^#^	ethanol	466.9	-	-	286.4	-	188.5	ppb	<0.005
			acetone	358.6	-	-	330.2	-	225.7		
			butane	90.3	-	-	25.8	-	56.2		
			dimethyl sulfide	11.9	-	-	9.2	-	9.3		
			isoprene	100.3	-	-	61.5	-	70.8		
			propanal	7.8	-	-	6.7	-	6.9		
			1-propanol	54.8	-	-	17.0	-	6.6		
			2-pentanone	7.5	-	-	5.3	-	4.8		
			furan	4.7	-	-	4.3	-	3.7		
			o-xylene	22.1	-	-	18.7	-	17.4		
			ethylbenzene	19.6	-	-	10.4	-	10.4		
			pentanal	5.9	-	-	0	-	0		
			hexanal	4.5	-	-	0	-	0		
			nonane	Not quantified							
8	Caldeira, [46]	asthma	6 proposed VOCs (Nq)	2,2,4,6,6-pentamethylheptane, 3,6-dimethyldecane, decane, dodecane, nonane, tetradecane							
9	Phillips, [14]	COPD	isoprene	97.6	92.3	-	-	-	96.5	Occurrence rate (%)	-
			acetic acid	92.2	96.2	-	-	-	94.7		
			benzaldehyde	100	100	-	-	-	100		
			benzene	100	98.7	-	-	-	100		
			carbon dioxide	100	100	-	-	-	100		
			hexanal	90.2	94.9	-	-	-	0		
			toluene	100	93.6	-	-	-	0		
			1-heptenal	46.3	1.3	-	-	-	0		
			sulphur dioxide	71.1	80.3	-	-	-	63.2		
			1,3,5-cycloheptatriene	4.9	28.2	-	-	-	0		
10	Buszewski, [44]	LC	acetone	44.2–53.45	-	-	34.57–390	-	-	ppb	<0.005
			benzene	1.38–14.97	-	-	1.29–3.82	-	-		<0.005
			butanal	1.35–1.87	-	-	1.32–2.55	-	-		<0.001
			2-butanone	1.35–3.18	-	-	1.35–2.86	-	-		<0.001
			ethyl acetate	1.12–8.22	-	-	3.98 -22.89	-	-		<0.001
			ethylbenzene	2.22–18.38	-	-	1.45–3.16	-	-		<0.001
			furan	1.67–3.25	-	-	1.53–2.81	-	-		<0.005
			2-pentanone	1.80–4.11	-	-	3.25–8.77	-	-		<0.001
			propanal	1.56–3.44	-	-	1.56–3.74	-	-		<0.001
			1-propanol	0	-	-	4.37–13.15	-	-		<0.001
			2-propanol	3.21–4.17	-	-	3.32–7.19	-	-		<0.001
			2-propenal	5.10–9.57			6.84–94.36				<0.005
11	Gahleitner, [65]	asthma	8 proposed markers (nq)	1-(methylsulfanyl)propane; octadecyne; 1,4-dichlorobenzene; 1,7-dimethylnaphtalene; 1-isopropyl-3-methylbenzene; 2-octenal; 4-isopropenyl-1-methylcyclohexene; ethylbenzene;							
12	Fu, [67]	LC ^#^	2-butanone	1.78–8.38	-	-	0.45–2.34	-		nmol/L	<0.001
			3-hydroxy-2-butanone	0.13–077	-	-	0.02–0.15	-	-		<0.001
			2-hydroxyacetaldehyde	0.23–1.13	-	-	0.03–0.45	-	-		<0.001
			4-hydroxyhexenal	0.005–0.05	-	-	0.007–0.09	-	-		<0.005
13	Bousamra, [68]	LC ^#^	2-butanone	~ 3.3	^-^	-	~ 1.8	-	-	nmol/L	<0.001
			3-hydroxy-2-butanone	~ 0.25	^-^	-	~ 0.1	-	-		<0.001
			2-hydroxyacetaldehyde	~ 0.3	^-^	-	~ 0.2	-	-		<0.001
			4-hydroxyhexenal	~ 0.3	^-^	-	~ 0.15	-	-		<0.001
14	Handa, [36]	LC	9 proposed markers (nq)	2-metylbutylacetat; 3-methyl-1-butanol; ethylbenzol; heptanal; hexanal; iso-propylamin; n-dodecane; cyclohexanone							<0.01 to <0.001
15	Ma, [69]	LC ^#^	Toluene	22.01–291.6	-	-	18.86–99.8	-	-	ng/L	-
			ethylbenzene	14–85.67	-	-	3.76–218.1	-	-		-
			p-xylene + m-xylene	9.33–82.5	-	-	0.82–55.39	-	-		-
			o-xylene	2.93–14.8	-	-	1.31–23.0	-	-		-
			isopropyl benzene	0.24–0.86	-	-	0.19–0.67	-	-		-
16	Zou, [70]	LC	5 proposed markers (nq)	2, 6, 11-trimethyl-dodecane; 5-(2-methyl-) propyl- nonane; hexadecanal; 8-hexyl- pentadecane; 2,6-di-tert-butyl-, 4-methyl- phenol							<0.001 to 0.022
17	Li, [71]	LC	2-butanone	~3.5	-	-	~0.8	-	~ 0.7	nmol/L	95% CI
			4-hydroxy-2-hexenal	~0.0005	-	-	~0.0003	-	~ 0.0003		
			3-hydroxy-2-butanone	~0.03	-	-	~0.01	-	~ 0.01		
			hydroxyacetaldehyde	~0.04	-	-	~0.02	-	~ 0.02		
			4-hydroxy-2-nonenal	~0.002	-	-	~0.001	-	~ 0.001		
			2-pentanone	~1.2	-	-	~0.9	-	~ 0.8		
18	Ma, [72]	LC ^#^	propanol	7415.3	-	-	1975.3	-	-	ng/L	-
			acetone	1811.6	-	-	579.9	-	-		-
			methanol	225	-	-	76.7	-	-		-
19	Schumer [73]	LC	2-butanone	3.4	2.47 ^	-	1.4	1.26 ^	-	nmol/L	-
			3-hydroxy-2-butanone	0.31	0.15 ^	-	0.09	0.07 ^	-		-
			2-hydroxyacetaldehyde	0.33	0.29 ^	-	0.17	0.019 ^	-		-
			4-hydroxyhexenal	0.007	0.007 ^	-	0.002	0.001 ^	-		-
20	Gaida A, [19]	COPD ^λ^	benzene	96–100	96	-	100	96 ^	-	Occurrence rate (%)	<0.005 to <0.02
			acetic acid	96–100	96–100	-	100	96–100 ^	-		
			toluene	100	96–100	-	100	100 ^	-		
			m,p-xylene	96–100	74–93	-	96-100	80–85 ^	-		
			1,6-dimethyl-1,3,5-heptatriene	74–100	7–19	-	40–71	0 ^	-		
			o-xylene	93–100	33–50	-	57–96	26–36 ^	-		
			1-ethyl-3-methyl benzene	89–100	33–61	-	60–92	26–28 ^	-		
			linalyl acetate	9–89	11–96	-	3–88	24–89 ^	-		
			tridecane	100	85–100	-	77–100	88–100 ^	-		
			phenole	100	96–100	-	100	100 ^	-		
			m/p-cresol	36–48	7–50	-	30–71	44–48 ^	-		
			indole	64–100	67–100	-	87–96	96–100 ^	-		
			vinyl acetate	96–100	7–9	-	57–88	0–8 ^	-		
			butanone	78–100	82–85	-	63–71	22–60 ^	-		
21	Allers, [37]	COPD	acetonitrile	~25	~1 ^	-	~33	~6 ^	-	Intensity (arbitral units)	=0.01
			2-butanone	~2.3	~1.9 ^	-	~3.1	~1.8			=0.05
			2-pentanone	Intensity not specified							-
22	Schallschmidt [75]	LC	n-hexane	1.3	0.8 ^˄^	-	2.1	0.7 ^˄^	-	ng/L	<0.001 to <0.05
			3-methylpentane	0.8	0.8 ^˄^	-	1.6	0.5 ^˄^	-		
			cyclohexane	1.5	1.9 ^˄^	-	4.4	4.8 ^˄^	-		
			n-nonanal	1.9	2.4 ^˄^	-	2.8	1.3 ^˄^	-		
			1-butanol	5.1	10.1	-	2.2	1.9 ^˄^	-		
			2-butanone	6.9	6.6	-	19.3 ^˄^	4.6 ^˄^	-		
			2-pentanone	2.9	2.7	-	10.8 ^˄^	3.0 ^˄^	-		
23	van Vliet, [76]	asthma	7 proposed markers (nq)	1, 2-dimethylcyclohexane; 2-ethylhexanal; 2-methylfuran; 6, 10-dimethyl-5,9-undecadien-2-one; nonanal; octanal							
24	Lamote, [78]	LC	6 proposed markers (nq)	diethyl ether, methylcyclopentane, nonanal, limonene, cyclohexan, isothiocyanatocyclohexane							
25	Jareno-Esteban, [79]	COPD	5 proposed markers (nq)	hexanal; heptanal; nonanal; propanoic acid; nonanoic acid							
26	Oguma, [80]	LC ^#^	Cyclohexane	0.1	-	-	0.2	-	-	ppb	=0.002
			Xylene	0.16	-	-	0.07	-	-	ppb	=0.0001
27	Sakumura, [16]	LC	5 proposed markers (nq)	hydrogen isocyanide; methanol; acetonitrile; isoprene; 1-propanol							
28	Pizzini, [81]	COPD ^#^	cyclohexanone	35	-	-	-	-	-	Occurrence rate (%)	<0.001 to =0.006
			n-butane	96	-	-	-	-	-		
			4-heptanone	48	-	-	-	-	-		
			2-pentanone	79	-	-	-	-	-		
			n-heptane	99	-	-	-	-	-		
			methyl propyl sulfide	77	-	-	-	-	-		
			dimethyl disulfide	93	-	-	-	-	-		
			6-methyl-5-heptene-2-one	98	-	-	-	-	-		
			2,4-dimethylheptane	90	-	-	-	-	-		
			2,6-dimethyloctane	70	-	-	-	-	-		
			cyclohexane	95	-	-	-	-	-		
			2-methylhexane	82	-	-	-	-	-		
29	Wang, [82]	LC	12 proposed markers (nq)	heneicosane; 3-ethyltoluene; 1,2,3-trimethylbenzene, N-propylbenzene; indan; methylcyclohexane 1-methyl-3 propylbenzene; propylcyclohexane; o-xylene; 4-methyl-2-pentanone; 5-methylindan;							<0.001
30	Cai, [41]	LC	23 proposed markers (nq)	dimethylmethane; ethanol; methane; isoprene; hexane; heptane methyl-cyclopentane; 2-methylheptane; octane; 3-methyloctane; 1,4-dimethylbenzene; ethenylbenzene; dodecane; tetradecane; tridecane; 2.2.4.6.6-pentamethyl-heptane; 2,5,5-trimethyl-2,6-heptadien-4-one; limonene; benzene;; 2-phenyl-propylbutyrate; 1,2,6-trimethylnaphthalene; 3-methylnonane;							

*—when a single value is reported, the concentration refers to mean range concentration detected in the investigated samples; #—smoking status of patients was not detailed with respect of obtained concentrations/values, consequently the quantified markers were placed in the first column (s) just in an aleatory way; ^—the value refers to the concentration measured in former + never smokers; λ—inter-laboratory comparison, different values reported are related to the discrepancies between the two investigated sited; pM^1^ = 10^−12^ M; LC- lung cancer; nq—not quantified.

## Data Availability

Data is contained within the article.
